# MYSM1 attenuates osteoarthritis by recruiting PP2A to deubiquitinate and dephosphorylate RIPK2

**DOI:** 10.1038/s41413-024-00368-y

**Published:** 2025-01-02

**Authors:** Kang Wei, Chuankun Zhou, Zixing Shu, Xingru Shang, Yi Zou, Wei Zhou, Huanhuan Xu, Yulin Liang, Tian Ma, Xuying Sun, Jun Xiao

**Affiliations:** 1 Department of Orthopedics, Tongji Hospital, Tongji Medical College, Huazhong University of Science and Technology, Wuhan, Hubei China; 2 Department of Plastic Surgery, Zhongnan Hospital of Wuhan University, Wuhan, China; 3 Institute of Hepatobiliary Diseases, Transplant Center, Hubei Key Laboratory of Medical Technology on Transplantation, Zhongnan Hospital of Wuhan University, Wuhan, China; 4 Department of Obstetrics and Gynecology, Wuhan Children’s Hospital, Tongji Medical College, Huazhong University of Science and Technology, Wuhan, China

**Keywords:** Pathogenesis, Bone

## Abstract

Osteoarthritis (OA), the most prevalent degenerative joint disease, is marked by cartilage degradation and pathological alterations in surrounding tissues. Currently, no effective disease-modifying treatments exist. This study aimed to elucidate the critical roles of Myb-like, SWIRM, and MPN domains 1 (MYSM1) and its downstream effector, Receptor-interacting protein kinase 2 (RIPK2), in OA pathogenesis and the underlying mechanisms. Our findings revealed reduced MYSM1 levels in the cartilage of OA patients and mouse models. Genetic or adenovirus-induced MYSM1 knockout exacerbated OA progression in mice, whereas MYSM1 overexpression mitigated it. Mechanistically, MYSM1 inhibited the NF-κB and MAPK signaling pathways. Conversely, downstream RIPK2 significantly increased OA-like phenotypes and activated the NF-κB and MAPK pathways. The *Ripk2*^*S176D*^ mutation accelerated OA pathogenesis, while *Ripk2* silencing or *Ripk2*^*S176A*^ mutation deactivated NF-κB and MAPK pathways, counteracting the role of MYSM1. MYSM1 deubiquitinates and dephosphorylates RIPK2^S176^ by recruiting protein phosphatase 2 A (PP2A). These results suggest that targeting MYSM1 or downstream RIPK2 offers promising therapeutic potential for OA.

## Introduction

Osteoarthritis (OA), the most prevalent age-related arthritis, impacts approximately one in three individuals over 65, imposing an increasing burden on families and society.^1,2^ The disease is characterized by progressive cartilage degradation, synovitis, subchondral bone remodeling, and osteophyte formation.^3–6^ OA currently lacks effective disease-modifying therapies. The underlying mechanisms of OA remain poorly understood. Inflammation is recognized as an early event in OA,^7^ initiating the activation of synovial cells surrounding the cartilage.^8,9^ This activation leads to the loss of cartilage matrix components, such as Aggrecan and collagen II,^10^ promotes the production of inflammatory cytokines, including interleukin-1β (IL-1β), and increases the expression of inflammatory proteins like cyclooxygenase-2 (COX-2) and nitric oxide synthase (iNOS).^11^ Additionally, it triggers the production of matrix-degrading enzymes, such as matrix metalloproteinases (MMPs) and a disintegrin and metalloproteinase with thrombospondin motifs (Adamts), all of which contribute to progressive cartilage degeneration and loss.^12^

Myb-like, SWIRM, and MPN domains 1 (MYSM1) have been reported to deubiquitinate TRAF3, TRAF6, and RIPK2.^13,14^ MYSM1 modulates the functions of hematopoietic stem cells, lymphocytes, and other blood cells,^15,16^ and is considered a potential target for preventing aging and age-associated diseases.^17^ RIPK2, regulated by MYSM1, is a kinase involved in the downstream signaling of nuclear oligomerization domain (NOD)-like receptors NOD1 and NOD2.^18,19^ The RIPK2^104Asp^ variant in animals leads to an exaggerated response to joint injury, predisposing them to OA.^20^ The involvement of MYSM1 in OA pathogenesis, however, has not been elucidated.

This study observed decreased MYSM1 expression in OA patients and in mice subjected to destabilized medial meniscus (DMM) surgery. Similarly, MYSM1 was downregulated in chondrocytes stimulated with IL-1β. Gain- and loss-of-function experiments identified MYSM1 as a modulator of chondrocyte degeneration and inflammation. MYSM1 deletion activated NF-κB and MAPK signaling, significantly promoting OA development and exacerbating cartilage degeneration. Furthermore, our study demonstrated that MYSM1 modulates chondrocyte degeneration through RIPK2^Ser176^ (RIPK2^S176^) phosphorylation. Specific RIPK2^S176^ mutations significantly affected NF-κB and MAPK signaling, reversing MYSM1’s anti-inflammatory response. Mechanistically, PP2A interacted with and dephosphorylated RIPK2 at S176, reducing RIPK2 ubiquitination. MYSM1 enhanced the interaction between PP2A and RIPK2, further deubiquitinating and amplifying PP2A’s role in dephosphorylating RIPK2 at S176. This study positions MYSM1 as a novel target for OA treatment and uncovers mechanisms targeting downstream RIPK2, suggesting that MYSM1 cellular pathways could offer promising therapeutic strategies for OA patients.

## Results

### Reduced MYSM1 expression during OA development

**Fig. 1:**
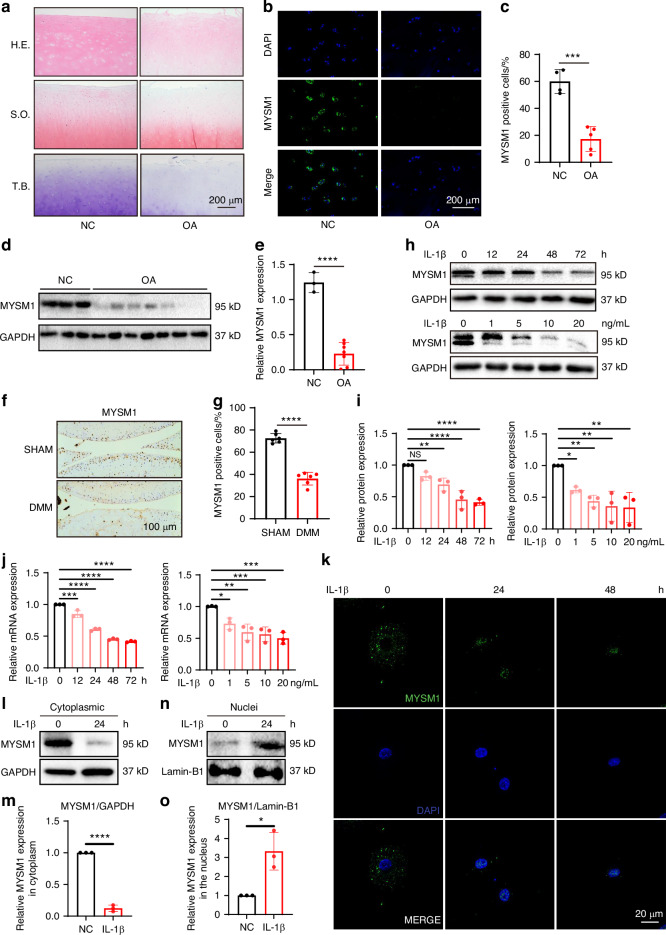
Reduced MYSM1 expression in human samples, animal models of OA, and in vitro settings. **a** Representative images of Hematoxylin-Eosin (H.E.), Safranine O/fast green (S.O.), and Toluidine blue (T.B.) staining in human samples. Scale bar = 200 μm. **b** MYSM1 staining in normal and OA cartilage. Scale bar = 200 μm, NC, *n* = 4; OA, *n* = 5. **c** Quantitative analysis of MYSM1 positive cells in (**b**). **d** Western blots showing MYSM1 expression in human cartilage, NC, *n* = 3; OA, *n* = 7. **e** Relative MYSM1 expression in (**d**). **f** MYSM1 staining showing decreased MYSM1 positive cells in DMM-operated mice compared to the Sham group. Scale bar = 100 μm. **g** Quantitative analysis of MYSM1 positive cells in (**f**). **h** Western blots showing reduced MYSM1 expression after treatment with different concentrations of IL-1β (0, 1, 5, 10, 20 ng/mL) for 48 h in primary mouse chondrocytes or 5 ng/mL IL-1β treatment for different time points (0, 12, 24, 48, 72 h) in primary mouse chondrocytes. **i** Relative MYSM1 expression in (**h**), *n* = 3. **j** Relative mRNA levels of *Mysm1* determined by qRT-PCR in primary mouse chondrocytes after stimulation with various doses of IL-1β for 24 h or 5 ng/mL IL-1β for different time points, *n* = 3. **k** Immunofluorescence of MYSM1 showing its expression and location after 5 ng/mL IL-1β treatment for 24 h or 48 h. Scale bar = 20 μm. **l** Western blots showing MYSM1 expression in the cytoplasm of mouse chondrocytes and (**m**) its quantitative analysis, *n* = 3. **n** Western blots showing MYSM1 expression in the nucleus of mouse chondrocytes and (**o**) its quantitative analysis, *n* = 3. All data are presented as mean ± SD. *P < 0.05, **P < 0.01, ***P < 0.001, ****P < 0.000 1, NS not significant

To investigate MYSM1’s role in OA pathogenesis, MYSM1 levels were examined in severe OA cartilage samples from patients undergoing joint replacement surgery, as well as in normal cartilage from individuals without bone diseases. OA cartilage samples exhibited more severe cartilage loss and a thinner cartilage layer compared to normal controls, as shown by H.E., S.O., and T.B. staining (Fig. 1a). Immunofluorescence (Fig. 1b, c) and western blots (Fig. 1d, e) confirmed reduced MYSM1 levels in OA patient cartilage. Human sample information and the proportion of MYSM1-positive cells are detailed in Tables S1–2. Additionally, MYSM1 expression was also decreased in mouse cartilage 8 weeks post-DMM surgery (Fig. 1f, g). To further explore MYSM1’s role in OA pathogenesis, primary chondrocytes from mice were cultured to assess MYSM1 changes in vitro. IL-1β was used to induce an OA inflammatory response.^21^ Western blots and RT-PCR analysis demonstrated a dose- (0, 1, 5, 10, 20 ng/mL) and time-dependent (0, 12, 24, 48, 72 h) reduction in MYSM1 following IL-1β exposure (Fig. 1h–j). MYSM1 was primarily localized in the cytoplasm; however, after 24 and 48 h of IL-1β treatment, MYSM1 significantly decreased and translocated to the nucleus (Fig. 1k). Western blot confirmed decreased cytoplasmic MYSM1 expression after IL-1 stimulation (Fig. 1l, m), whereas nuclear MYSM1 increased (Fig. 1n, o). These results suggest that MYSM1 downregulation and translocation are associated with OA development.

### MYSM1 regulated chondrocyte degeneration induced by IL-1β

The findings prompted an investigation into MYSM1’s involvement in cartilage degeneration. Chondrocytes were treated with 5 ng/mL IL-1β to mimic OA pathogenesis in vitro. This treatment significantly elevated cartilage catabolism factors such as MMP3, MMP13, ADAMTS4, ADAMTS5, and inflammatory markers COX2 and iNOS. Conversely, IL-1β notably suppressed anabolism markers Aggrecan and Collagen II, indicating a successful induction of an OA-like phenotype in chondrocytes.

Interestingly, overexpression of *Mysm1*reduced catabolism and inflammatory markers at both mRNA and protein levels (Fig. S1A–H). Additionally, *Mysm1*overexpression enhanced anabolism factors *Aggrecan* and *Collagen II*, although these factors remained unchanged in the presence of IL-1β (Fig. S1G, H). In contrast, chondrocytes isolated from *Mysm1*-KO mice exhibited increased cartilage catabolism and inflammation markers when exposed to IL-1β (Fig. S1I–P) compared to control chondrocytes. While IL-1β-induced downregulation of *Aggrecan* and *Collagen II* was not significantly affected in *Mysm1*-KO chondrocytes, these anabolism factors were significantly reduced in *Mysm1*-KO chondrocytes without IL-1β treatment (Fig. S1I, O-P). Similarly, silencing *Mysm1* with si-*Mysm1* enhanced IL-1β-induced levels of MMP3, MMP13, ADAMTS4, ADAMTS5, COX2, and iNOS (Fig. S2A–F). These findings suggest that MYSM1 exerts its protective role primarily by suppressing cartilage catabolism factors and ameliorating the inflammatory response in chondrocytes.

### *Mysm1* regulates OA development in mice

The possible association between MYSM1 and OA was further examined in vivo. Mice received intra-articular injections of Sh-*Con* or Sh-*Mysm1* adenoviruses once a week for 8 weeks in both sham-operated and DMM groups. MYSM1 staining confirmed the efficiency of *Mysm1* knockdown in the cartilage (Fig. S2G-H). Moderate cartilage destruction was observed post-DMM surgery, and S.O. and T.B. staining revealed that *Sh-Mysm1* significantly exacerbated cartilage destruction, evidenced by a higher OARSI score 8 weeks post-DMM (Fig. S2I-J). MicroCT scans indicated increased osteophyte formation around the joints in mice injected with Sh-*Mysm1* viruses (Fig. S2K-L). Additionally, MMP3 and MMP13 positive cells were significantly increased in the Sh-*Mysm1* group compared to the *Sh-Con* group post-DMM surgery (Fig. S2M).

**Fig. 2:**
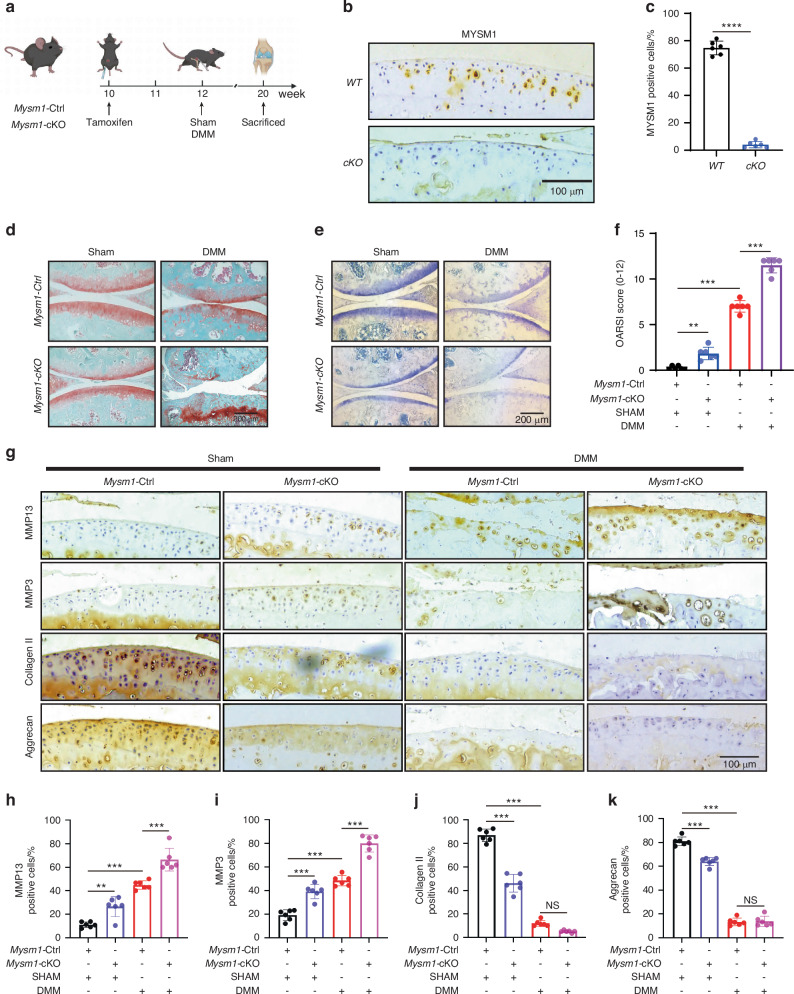
Deletion of *Mysm1* in mouse chondrocytes promoted cartilage destruction at 8 weeks post-DMM surgery. **a** Schematic illustration of tamoxifen administration and DMM surgery for *Mysm1*-Ctrl and *Mysm1*-cKO mice, created with BioRender. **b** IHC staining of MYSM1 in *Mysm1*-cKOor WT mice cartilage 8 weeks after DMM surgery, scale bar = 100 µm. **c** Quantitative analysis of MYSM1 in (**b**), *n* = 6. **d** S.O. and (**e**) T.B. staining, and (**f**) OARSI grade of knee joints from *Mysm1*-cKO or WT mice 8 weeks post-DMM surgery. Scale bars = 200 µm, *n* = 6. **g** IHC staining of MMP13, MMP3, Collagen II, and Aggrecan. Scale bar = 100 µm, *n* = 6. Quantification of (**h**) MMP13, (**i**) MMP3, (**j**) Collagen II, and (**k**) Aggrecan in joint cartilage of *Mysm1*-cKO or *Mysm1*-Ctrl mice 8 weeks post-DMM surgery, *n* = 6. **P < 0.01, ***P < 0.001, ****P < 0.000 1, NS not significant

To specifically delete the *Mysm1* gene in chondrocytes, *Mysm1*-Ctrl and *Mysm1*-Cko mice were used. The procedure is outlined in Fig. 2a. Eight weeks post-surgery, IHC staining showed a dramatic reduction of MYSM1-positive cells in the cartilage of *Mysm1*-cKO mice (Fig. 2b, c). *Mysm1*-cKO mice exhibited severe cartilage loss and higher OARSI scores compared to *Mysm1*-Ctrl mice in both sham-operated and DMM groups (Fig. 2d–f). Histomorphometry results indicated that *Mysm1* deletion enhanced MMP13 and MMP3 positive chondrocytes in both sham and DMM groups (Fig. 2g–i). While Collagen II and Aggrecan positive chondrocytes remained unchanged in *Mysm1*-cKO mice post-DMM surgery, they decreased in *Mysm1*-cKO sham-operated mice (Fig. 2g, j, k). Additionally, *Mysm1*-cKO mice exhibited more osteophyte formation in MicroCT images (Fig. S3A-B). To overexpress *Mysm1* in cartilage, mice were administered intra-articular injections of AV-*con* or *AV-Mysm1 virus* once a week for 8 weeks in both sham-operated and DMM groups (Fig. S3C). IHC staining confirmed MYSM1 overexpression in the cartilage (Fig. S3D-E). The AV-*Mysm1* group demonstrated a significant reduction in cartilage deterioration, as evidenced by S.O. and T.B. staining (Fig. S3F), with lower OARSI grades 8 weeks post-DMM surgery (Fig. S3G). Additionally, the AV-*Mysm1* group showed a significant increase in Aggrecan and a decrease in MMP13 levels compared to the DMM group (Fig. S3H-I). These results suggest that MYSM1 significantly alleviates OA progression, while MYSM1 deletion promotes cartilage degeneration and increases osteophyte formation in mice post-DMM in vivo.

### MYSM1 attenuates NF-κB and MAPK signaling pathways in chondrocytes

**Fig. 3:**
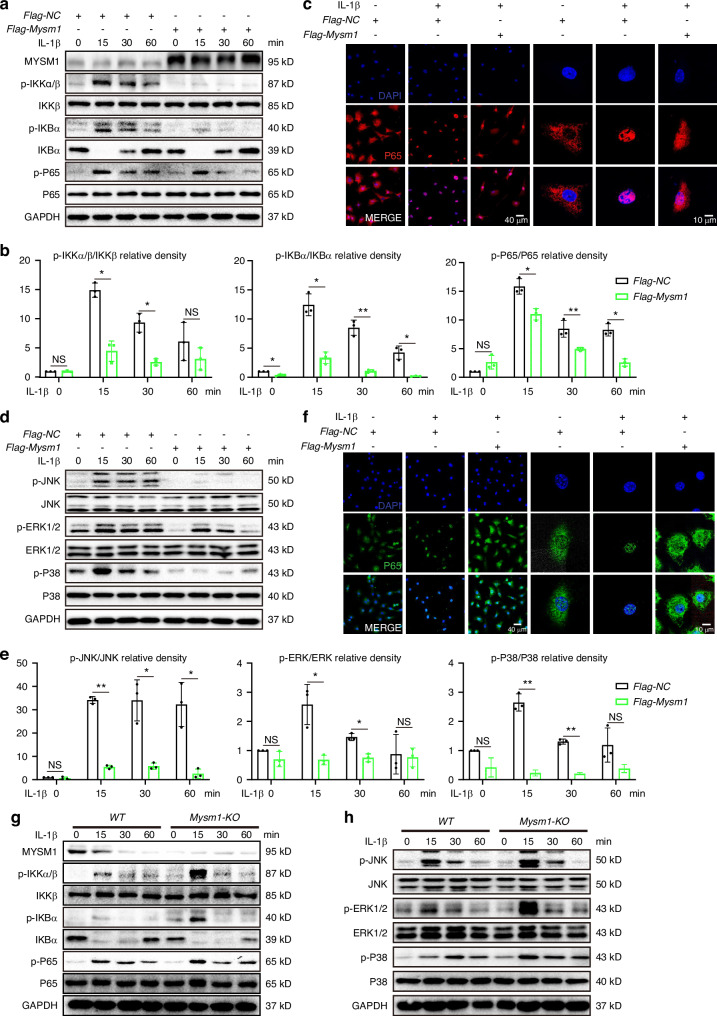
MYSM1 regulated NF-κB and MAPK signaling pathways. **a** Representative Western blot of NF-κB signaling pathways in primary mouse chondrocytes overexpressing *Mysm1* and (**b**) the phosphorylation ratio, *n* = 3. **c** Immunofluorescence staining of P65 in primary chondrocytes after treatment with 5 ng/mL IL-1β for 15 minutes. Scale bar = 40 and 10 µm. **d** Representative Western blot of MAPK signaling pathways in chondrocytes overexpressing *Mysm1* and (**e**) the phosphorylation ratio, *n* = 3. **f** Immunofluorescence staining of JNK in primary mouse chondrocytes after treatment with 5 ng/mL IL-1β for 15 minutes, scale bar = 40 and 10 µm. **g** Representative Western blot of NF-κB signaling pathways in control and *Mysm1*-KO chondrocytes. **h** Representative Western blot of MAPK signaling pathways in control and *Mysm1*-*KO* chondrocytes. All data are presented as mean ± SD. *P < 0.05, **P < 0.01, NS not significant

Accumulating data demonstrate that NF-κB and MAPK are pivotal signaling pathways involved in OA development, activated in degenerated chondrocytes during aging and inflammation.^22,23^ To investigate whether MYSM1 influences these pathways, western blot analysis was conducted. MYSM1 overexpression significantly decreased the phosphorylation levels of IKKα/β, P65, IKBα, JNK, ERK, and P38 under IL-1β treatment at various time points (Fig. 3a, b, d, e). Additionally, immunofluorescence showed that P65 and JNK translocated to the nucleus following IL-1β treatment, but this translocation was inhibited by MYSM1 overexpression (Fig. 3c, f). Similar patterns were observed with ERK and p38 in response to IL-1β and MYSM1 overexpression (Fig. S4A-B). In contrast, chondrocytes from *Mysm1*-KO mice exhibited increased expression of phosphorylated IKKα/β, P65, IKBα, JNK, ERK, and P38 when exposed to IL-1β (Fig. 3g, h, Fig. S4C). To confirm MYSM1’s specific role in these signaling pathways, *Mysm1* plasmids were reintroduced into *Mysm1*-KO chondrocytes, significantly restoring the phosphorylation levels of IKKα/β, P65, IKBα, JNK, ERK, and P38 (Fig. S4D-E). These results indicate that MYSM1 deactivates NF-κB and MAPK signaling pathways, thereby protecting mouse chondrocytes against inflammation.

### MYSM1 affects the NOD-signaling pathway by regulating RIPK2 dephosphorylation and ubiquitination

**Fig. 4:**
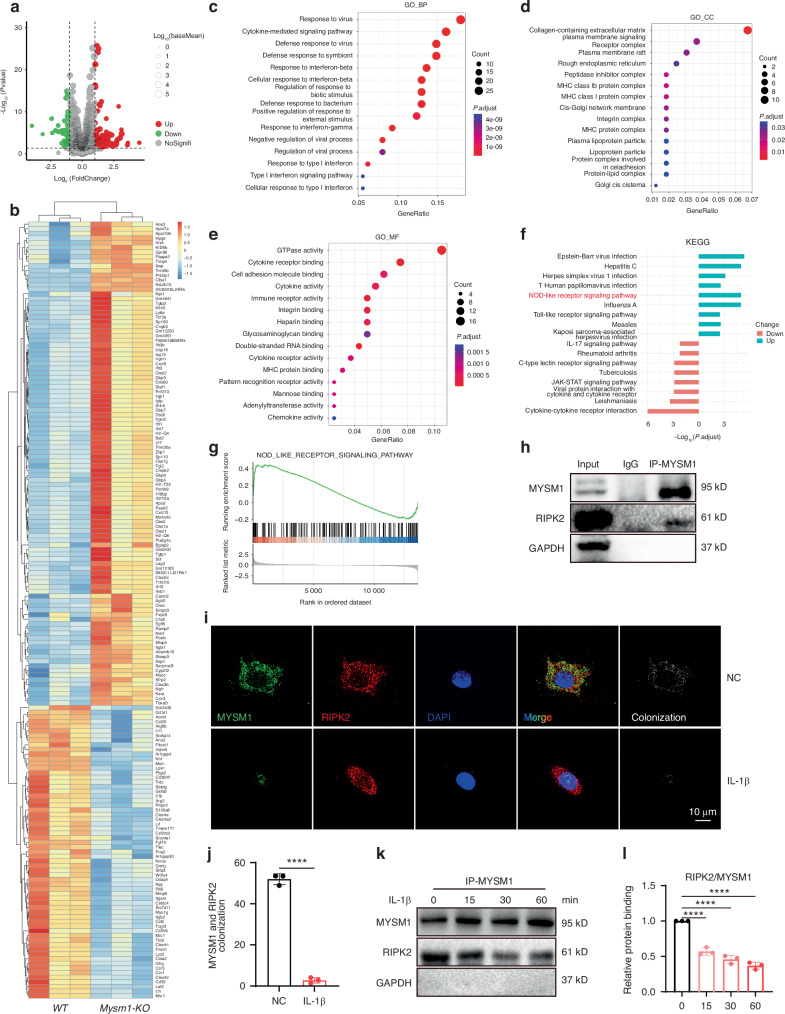
RNA sequencing and bioinformatics analysis revealed the function of *Mysm1* in chondrocytes. **a** The volcano plot displays 104 upregulated DEGs and 63 downregulated genes. **b** The heatmap illustrates the up- and down-regulated genes. **c** Enrichment analysis of biological processes from the GO database. **d** Enrichment analysis of cellular components from the GO database. **e** Enrichment analysis of molecular functions from the GO database. **f** KEGG enrichment analysis of key pathways regulated by MYSM1. **g** GSEA of DEGs. **h** RIPK2 was co-immunoprecipitated with MYSM1. **i** Immunofluorescence staining of RIPK2 and MYSM1, scale bars = 200 µm, and (**j**) its quantitative analysis, *n* = 3. **k** The association between RIPK2 and MYSM1 after IL-1β induction for 15, 30, and 60 minutes, and (**l**) its quantitative analysis, *n* = 3. All data are presented as mean ± SD. ****P < 0.000 1

To further elucidate the molecular mechanisms underlying MYMS1 deficiency-induced chondrocyte degeneration, bulk RNA sequencing was performed on *Mysm1*-KO and control chondrocytes. Differentially expressed genes (DEGs) were identified using stringent criteria (logFC >1, *P* < 0.05) (Fig. 4a). A total of 104 upregulated and 63 downregulated genes were identified (Fig. 4b). GO enrichment analysis of DEGs indicated significant involvement in immune response and collagen-containing extracellular matrix processes (Fig. 4c–e). KEGG and GSEA analyses confirmed that the NOD-like receptor signaling pathway was markedly enhanced in *Mysm1-KO* chondrocytes (Fig. 4f, g), a pathway implicated in OA pathogenesis.^24^ NOD signaling activates NF-κB and MAPK pathways, which subsequently trigger the transcription of pro-inflammatory cytokines.^25^ RIPK2 plays a pivotal role in NOD-mediated downstream effects,^26^ being recruited by NOD-like receptors and involved in innate immunity, inflammation, and infection,^27,28^ and can be activated through phosphorylation.^29,30^ Thus, it was hypothesized that MYSM1 protects chondrocytes from degeneration by targeting RIPK2. In mouse primary chondrocytes, MYSM1 and RIPK2 interaction was confirmed *via* co-immunoprecipitation (Fig. 4h), and co-localization of these proteins was observed (Fig. 4i–j). However, IL-1β treatment weakened the MYSM1-RIPK2 interaction (Fig. 4k, l).

**Fig. 5:**
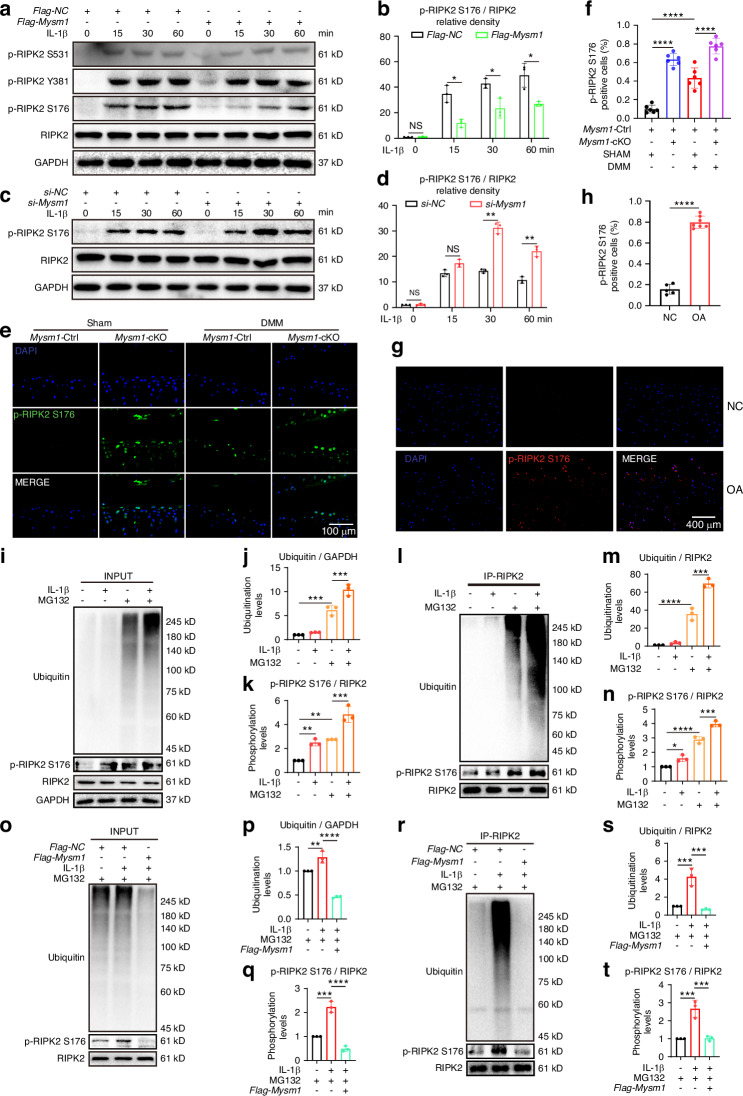
MYSM1 regulated RIPK2 phosphorylation and ubiquitination in primary mouse chondrocytes. **a** Representative Western blots of p-RIPK2 at S176, Y381, S531, and total RIPK2 expression. **b** The phosphorylation ratio of RIPK2 after *Mysm1* overexpression, *n* = 3. **c** Representative Western blots of p-RIPK2^S176^, RIPK2 expression, and (**d**) the phosphorylation ratio after *Mysm1* knockdown, *n* = 3. **e** The level of p-RIPK2^S176^in *Mysm1*-cKO or WT mice, scale bar = 100 µm. **f** The proportion of p-RIPK2^S176^positive cells in mouse cartilage, *n* = 6. **g** The level of p-RIPK2^S176^in human cartilage from normal and OA patients, scale bar = 400 µm, NC, *n* = 5; OA, *n* = 7. **h** The proportion of p-RIPK2^S176^positive cells in human cartilage. Mouse chondrocytes were pre-treated with 10 μmol/L MG132 for 2 hours followed by treatment with IL-1β at 5 ng/mL for 6 hours. **i** Representative Western blots of input ubiquitin, p-RIPK2^S176^, and RIPK2, and (**j**) quantification analysis of RIPK2 ubiquitination level, *n* = 3. **k** Quantification analysis of RIPK2 phosphorylation level, *n* = 3. **l** Immunoprecipitation assay using an anti-RIPK2 antibody to detect ubiquitination of RIPK2 and p-RIPK2 in mouse chondrocytes after IL-1β treatment, and (**m**) quantification analysis of RIPK2 ubiquitination level, *n* = 3. **n** Quantification analysis of RIPK2 phosphorylation level, *n* = 3. **o** Representative Western blots of input ubiquitin, p-RIPK2^S176^, and RIPK2 after *Mysm1* overexpression with IL-1β treatment, and (**p**) quantification analysis of RIPK2 ubiquitination level, *n* = 3. **q** Quantification analysis of RIPK2 phosphorylation level, *n* = 3. **r** Immunoprecipitation assay using anti-RIPK2 antibody to detect ubiquitination of RIPK2 and p-RIPK2 in mouse chondrocytes after *Mysm1* overexpression with IL-1β treatment, and (**s**) quantification analysis of RIPK2 ubiquitination level, *n* = 3. **t** Quantification analysis of RIPK2 phosphorylation level, *n* = 3. All data are presented as mean ± SD. *P < 0.05, **P < 0.01, ***P < 0.001, ****P < 0.000 1, NS not significant

Phosphorylation of RIPK2 at different sites was further examined, revealing that p-RIPK2 S176 decreased with MYSM1 overexpression, while p-RIPK2 S531 and p-RIPK2 Y381 remained unchanged (Fig. 5a, b). Conversely, p-RIPK2 S176 levels increased with *Mysm1* deletion (Fig. 5c, d), although total RIPK2 levels were unaffected by *Mysm1* regulation. In vivo, p-RIPK2 S176 positive cells were more numerous in DMM surgical and *Mysm1*-cKO mice (Fig. 5e, f), and cartilage from OA patients exhibited high levels of p-RIPK2 S176 (Fig. 5g, h). RIPK2 ubiquitination is essential for NOD interaction and subsequent NF-κB activation.^19,31,32^ Ubiquitination levels were assessed under IL-1β treatment with MG132, a proteasome inhibitor used for ubiquitination detection. Enhanced total protein and RIPK2 ubiquitination levels, along with increased pS176 levels, were observed following IL-1β exposure in the presence of MG132 (Fig. 5i–n). However, MYSM1 overexpression reduced both total and RIPK2 ubiquitination and p-RIPK2 S176 levels induced by IL-1β (Fig. 5o–t). These results suggest that MYSM1 intercepts the NOD pathway by reducing RIPK2 phosphorylation at S176 and ubiquitination in IL-1β-stimulated chondrocytes.

### RIPK2 accelerates chondrocytes degeneration and activates NF-κB and MAPK signaling pathways

Alterations in RIPK2 were examined for their impact on chondrocyte degeneration. Overexpressing *Ripk2* significantly elevated MMP3, MMP13, COX2, and iNOS levels induced by IL-1β in mouse chondrocytes (Fig. S5A-B). Conversely, knockdown of *Ripk2* reduced these markers (Fig. S5C-D). Application of the selective RIPK2 antagonist GSK583^33^ reversed chondrocyte degeneration induced by *Mysm1* knockdown (Fig. S5E-F), highlighting RIPK2 as a downstream modulator of MYSM1 whose absence exacerbates cartilage degeneration. Subsequent analysis of NF-κB and MAPK signaling pathways in chondrocytes revealed enhanced RIPK2^S176^ phosphorylation and ubiquitination following IL-1β treatment, further augmented by Ripk2 overexpression (Fig. S5G-J). NF-κB and MAPK signaling were also upregulated with *Ripk2* overexpression (Fig. S5K-L). Co-transfection of *Mysm1* and *Ripk2* in chondrocytes resulted in reduced p-RIPK2 levels (Fig. S6A-B), accompanied by suppression of over-activated NF-κB and MAPK signaling pathways (Fig. S6C-D). These results confirm that RIPK2 operates downstream of MYSM1, exacerbating inflammation and chondrocyte degeneration in mice.

### RIPK2 S176 phosphorylation is essential in activating NF-κB and MAPK signaling pathways

The findings above prompted inquiry into whether RIPK2 phosphorylation is pivotal for subsequent activation of NF-κB and MAPK. Employing the *Ripk2*^*S176D*^ mutation plasmid, sustained phosphorylation of RIPK2 was achieved at S176 (Fig. S7A-B). Notably, *Ripk2*^*S176D*^ mutation markedly enhanced NF-κB and MAPK signaling pathways compared to wild-type *Ripk2*, evidenced by elevated levels of p-IKKα/β, p-P65, p-IKBα, p-JNK, p-ERK, and p-P38 (Fig. S7C-D). In contrast, *Ripk2*^*S176A*^, which abolishes RIPK2 phosphorylation (Fig. S8A-B), reduced levels of p-IKKα/β, p-P65, p-IKBα, p-JNK, p-ERK, and p-P38 (Fig. S8C-D). Furthermore, overexpression of *Mysm1* failed to inhibit phosphorylation at pS176 (Fig. S9A-B) and did not negate the impact of *Ripk2*^*S176D*^ in activating NF-κB and MAPK pathways (Fig. S9C-D). Even after *Mysm1* overexpression, *Ripk2*^*S176D*^ mutation continued to activate NF-κB and MAPK pathways (Fig. S9E-F). Thus, sustained phosphorylation by *Ripk2*^*S176D*^ appeared to counteract MYSM1’s role in deactivating NF-κB and MAPK signaling. These results underscore the significance of S176 phosphorylation in RIPK2 for NF-κB and MAPK activation, highlighting MYSM1’s protective effect through RIPK2^S176^ dephosphorylation.

### MYSM1 recruits PP2A to facilitate RIPK2 deubiquitination and dephosphorylation

**Fig. 6:**
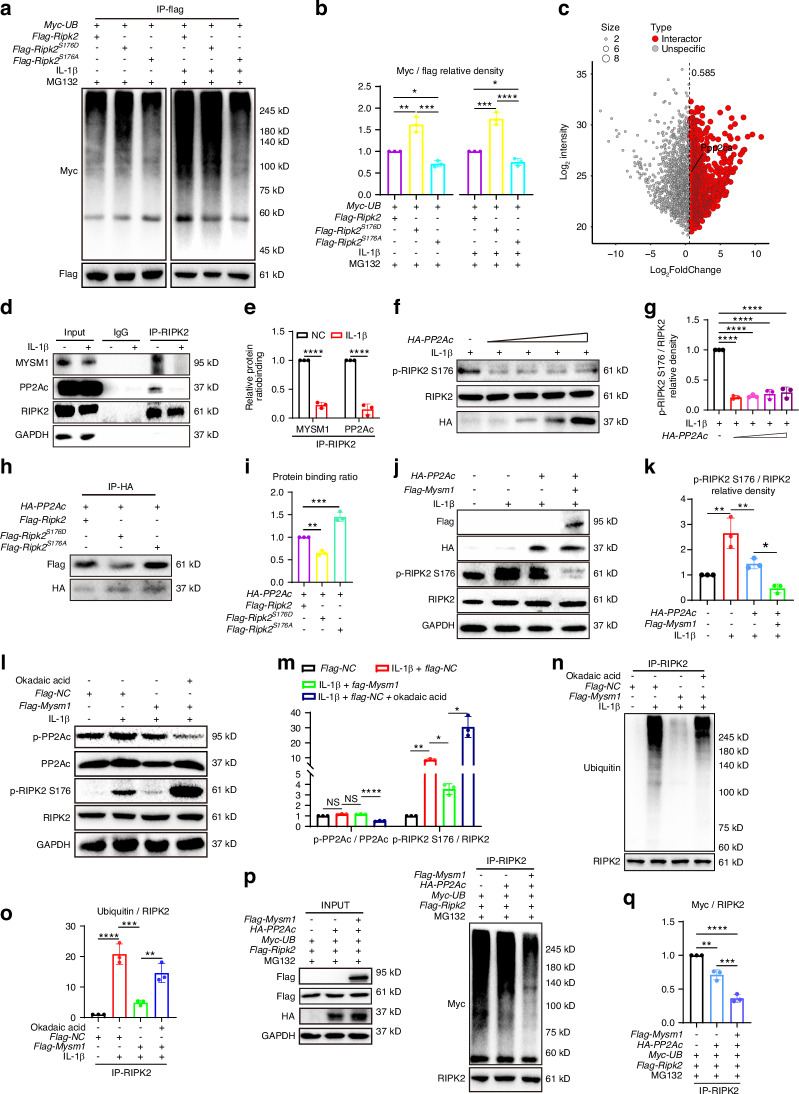
MYSM1 interacted with PP2A and promoted RIPK2 deubiquitination and dephosphorylation. **a** 293 T cells were transfected with *Myc-UB*, *Ripk2*, *Ripk2*^*S176D*^, or *Ripk2*^*S176A*^ plasmids for 48 hours, followed by treatment with 5 ng/mL IL-1β for 6 hours and pretreatment with 10 μmol/L MG132 for 2 hours. Immunoprecipitation assay showing RIPK2 ubiquitination, and (**b**) quantification of RIPK2 ubiquitination levels, *n* = 3. **c** Volcano plot displaying proteins interacting with MYSM1 identified through IP-MS. **d** Immunoprecipitation assay using anti-RIPK2 antibody to detect PP2Ac and MYSM1 interaction with RIPK2 with or without IL-1β exposure for 6 hours in chondrocytes, and (**e**) relative protein binding ratio, *n* = 3. **f** Western blots showing endogenous levels of p-RIPK2 and RIPK2 with increasing PP2Ac plasmid transfection in the presence of 5 ng/mL IL-1β for 15 minutes in mouse chondrocytes, and (**g**) the phosphorylation ratio, *n* = 3. (**h**) Immunoprecipitation assay of exogenous RIPK2, RIPK2^S176A^, and RIPK2^S176D^ interaction with PP2Ac in mouse chondrocytes, and (**i**) relative protein binding ratio, *n* = 3. **j** Western blots of p-RIPK2 and RIPK2 in chondrocytes transfected with*PP2Ac*, *Mysm1*, or control plasmids for 48 hours with 5 ng/mL IL-1β treatment for 15 minutes, and (**k**) the phosphorylation ratio, *n* = 3. **l** Western blots of p-PP2Ac, PP2Ac, p-RIPK2, and RIPK2 in mouse chondrocytes transfected with *Mysm1*with or without Okadaic Acid treatment, and (**m**) the phosphorylation ratio of p-PP2Ac and p-RIPK2, *n* = 3. **n** Immunoprecipitation assay showing RIPK2 ubiquitination after Okadaic Acid treatment, and (**o**) quantification of RIPK2 ubiquitination levels, *n* = 3. Cultured mouse chondrocytes were transfected with *Myc-UB*, *Pp2ac*, *Mysm1*, or control plasmids for 48 hours. **p** Immunoprecipitation assay using anti-FLAG antibody to detect RIPK2 ubiquitination level, and (**q**) quantification of RIPK2 ubiquitination levels, *n* = 3. All data are presented as mean ± SD. *P < 0.05, **P < 0.01, ***P < 0.001, ****P < 0.000 1

**Fig. 7:**
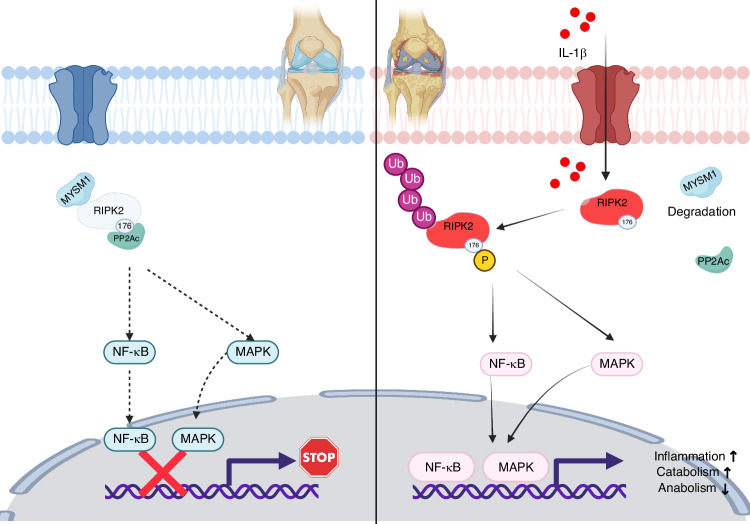
Schematic diagram depicting MYSM1-mediated recruitment of PP2A and regulation of RIPK2 deubiquitination and dephosphorylation in OA pathogenesis

Sustained phosphorylation of RIPK2 by *Ripk2*^*S176D*^ plasmids showed a high level of RIPK2 ubiquitination. Reduction of RIPK2 phosphorylation by *Ripk2*^*S176A*^ exhibited a low level of RIPK2 ubiquitination (Fig. 6a, b), indicating that pS176 of RIPK2 correlates with RIPK2 ubiquitination. However, as a deubiquitinating enzyme, the mechanism through which MYSM1 regulates RIPK2 phosphorylation remains unknown. Using immunoprecipitation and mass spectrometry analysis, we identified multiple potential MYSM1-binding proteins. One of these proteins, Ppp2ca (PP2Ac), functions as a serine/threonine protein phosphatase responsible for a significant portion of eukaryotic protein dephosphorylation events (Fig. 6c).^34^ The PP2A core enzyme comprises a scaffold subunit (A or PR65 subunit) and a catalytic subunit (C subunit, PP2Ac).^35^ We observed that PP2Ac interacted with RIPK2, but this interaction was abolished upon IL-1β treatment, and the binding of MYSM1 to RIPK2 was also disrupted under IL-1β stimulation (Fig. 6d, e). PP2Ac dose-dependently dephosphorylated RIPK2 at S176 (Fig. 6f, g). In terms of the RIPK2 and PP2Ac interaction, RIPK2^S176D^ reduced while RIPK2^S176A^ increased the binding of RIPK2 and PP2Ac (Fig. 6h, i). Interestingly, MYSM1 notably enhanced the role of PP2A in dephosphorylating RIPK2, as indicated by the pS176 levels (Fig. 6j, k). Okadaic Acid, a potent PP2A inhibitor, was used to inhibit PP2Ac by dephosphorylating PP2A.^36^ P-PP2Ac (Tyrosine 307, Y307) decreased after 10 μmol/L Okadaic Acid treatment. Although MYSM1 inhibits the pS176 level of RIPK2, it rapidly increased after exposure to Okadaic Acid (Fig. 6l, m). Okadaic Acid also significantly increased RIPK2 ubiquitination (Fig. 6n, o). Additionally, overexpression of PP2A in combination with MYSM1 induced even greater deubiquitination of RIPK2 (Fig. 6p, q). These results suggest that the phosphorylation of RIPK2^S176^ is essential for RIPK2 ubiquitination, and MYSM1 reduces the phosphorylation and ubiquitination of RIPK2 with the assistance of PP2A (Fig. 7).

## Discussion

OA is a prevalent joint disease predominantly affecting middle-aged and elderly individuals. Inflammatory changes within the joint are considered pivotal in the pathogenesis of OA.^37,38^ MYSM1 deficiency leads to heightened inflammation characterized by increased cytokine production.^13,14^ This study initially quantified MYSM1 levels in cartilage. We observed decreased MYSM1 expression in the articular cartilage of OA patients and in a mouse model of DMM, suggesting a protective role of MYSM1 against cartilage degeneration. Notably, overexpression of *Mysm1* reversed cartilage degeneration, whereas genetic deletion of *Mysm1* or *si-Mysm1* significantly exacerbated IL-1β-induced cartilage destruction in mouse primary chondrocytes. These results confirm that MYSM1 modulates OA pathogenesis. Furthermore, intra-articular injection of *Sh-Mysm1* adenoviruses after DMM surgery significantly aggravated cartilage degeneration compared to control adenovirus injection, while AV-*Mysm1* injection provided significant protection, highlighting the potential therapeutic role of MYSM1 in OA in vivo. To exclude adenoviral off-target effects, *Mysm1*^*fl/fl*^-cKO mice were employed in our study, revealing more severe cartilage destruction and osteophyte formation compared to *Mysm1*^*fl/fl*^-Ctrl mice. Interestingly, MYSM1 induced significant changes in catabolic factors such as MMP3 and MMP13, whereas anabolic factors like Collagen II and Aggrecan were minimally affected post-surgery. This suggests that while DMM surgery accelerates knee joint degeneration, *Mysm1* deletion may not further reduce anabolic factors under inflammatory conditions. These results underscore that reduced MYSM1 levels accelerate cartilage degeneration.

Inflammatory responses play a critical role in OA pathogenesis.^39–41^ Our findings demonstrate that *Mysm1* overexpression deactivates NF-κB and MAPK signaling pathways, whereas *Mysm1* knockdown significantly activates these pathways in mouse chondrocytes. RIPK2, a key mediator in NOD signaling associated with various chronic inflammatory conditions,^42^ was found in our study to interact with MYSM1 and undergo decreased phosphorylation and ubiquitinationin mouse chondrocytes.

Single IL-1β incubation induced RIPK2^Ser176^ phosphorylation and ubiquitination, suggesting these modifications as indicators of inflammation. RIPK2 overexpression or *Ripk2*^*Ser176D*^ mutation induced cartilage degeneration and activated NF-κB and MAPK pathways, while *Ripk2*^*Ser176A*^ mutation restrained these pathways compared to wildtype *Ripk2*, highlighting the regulatory role of RIPK2 phosphorylation in inflammation and OA pathogenesis. Moreover, MYSM1 failed to reverse NF-κB and MAPK activation caused by *Ripk2*^*Ser176D*^ mutation. These observations clarify that MYSM1 protects chondrocytes from inflammation through both deubiquitination and dephosphorylation of RIPK2 at the S176 site. Dephosphorylated and deubiquitinated RIPK2 downstream of MYSM1 maintains articular cartilage homeostasis by antagonizing NF-κB and MAPK signaling in OA.

As a deubiquitinase, MYSM1 may not directly dephosphorylate RIPK2; instead, it likely recruits other phosphatases during inflammation. Our results demonstrate that PP2Ac interacted functionally with RIPK2 and dephosphorylated it at the S176 site, an interaction impaired upon IL-1β exposure. PP2A plays a pivotal role in development, cell proliferation, cytoskeleton dynamics, and signaling pathway regulation.^43,44^ MYSM1 recruits PP2A to facilitate RIPK2 dephosphorylation, thereby promoting RIPK2^Ser176^ dephosphorylation and deubiquitination. Previous studies have shown that RIPK2 S176 autophosphorylation limits NOD2-driven cytokine responses,^30,45^ and RIPK2 polyubiquitination is essential for the NOD2 pathway and NF-κB activation.^46^ However, reciprocal regulation between RIPK2 ubiquitination and phosphorylation remains poorly understood. Our study reveals that the deubiquitinase MYSM1 interacts with and deubiquitinates RIPK2, recruiting PP2A to dephosphorylate RIPK2 and ultimately deactivate NF-κB and MAPK signaling pathways, thereby protecting chondrocytes from degeneration.

## Conclusions

This study conclusively demonstrated a reduction in MYSM1 expression in human OA cartilage, DMM mice cartilage, and IL-1β-treated chondrocytes. Furthermore, the absence of MYSM1 exacerbated OA pathogenesis by enhancing RIPK2 ubiquitination and phosphorylation. This research established a connection between the ubiquitination and phosphorylation of RIPK2 and pinpointed S176 of RIPK2 as a pivotal element in inflammation and cartilage degradation. Mechanistically, MYSM1 deubiquitinates and dephosphorylates RIPK2^S176^ by recruiting PP2A. These findings offer unprecedented evidence of the MYSM1/PP2A/RIPK2 axis’s role in preserving cartilage homeostasis, indicating that targeting MYSM1 and downstream RIPK2 could be therapeutically beneficial for OA treatment.

## Materials and methods

### Reagents and antibodies

Recombinant mouse IL-1β was sourced from R&D Systems (401-ML-005/CF, Minneapolis, MN, USA). Lipofectamine 3000 was obtained from Invitrogen (L3000008, Waltham, MA, USA). Secondary antibodies were procured from Jackson ImmunoResearch Laboratories (West Grove, PA, USA). MG132 (M8699) and the RIPK2 antagonist GSK583(SML1960) were purchased from Sigma (St. Louis, MO, USA). Okadaic acid (HY-N6785) was acquired from MedChemExpress (Shanghai, China).

### Human samples

Normal cartilage samples were obtained from cadaver donors without bone diseases at Zhongnan Hospital in Wuhan, China. OA cartilages were collected from severe OA patients who underwent joint replacement at Tongji Hospital in Wuhan, with informed consent obtained from all participants. The collection of human cartilage was approved by the Ethics Committee of Tongji Hospital, Tongji Medical College, Huazhong University of Science and Technology (No. TJ-IRB20210127). Clinical information of the donors is listed in Table S1.

### Animal model

*Mysm1*^*fl/fl*^ (*Mysm1*-Ctrl) mice were generously provided by Dr. Xiaoxia Jiang from the Institute of Military Cognition and Brain Sciences, Academy of Military Medical Sciences, Beijing. *Col2α1-Cre*^*ERT*^ mice were purchased from Jackson Laboratory. *CAG-Cre* mice were a generous gift from Dr. Ke Chen in the Department of Urology, Tongji Hospital, Tongji Medical College, Huazhong University of Science and Technology. *Mysm1* global knockout mice (*Mysm1*-KO) were generated by crossing *Mysm1*^*fl/fl*^ with *CAG-Cre* mice. *Mysm1* chondrocyte knockout mice (*Mysm1*-cKO) were generated by crossing *Mysm1*^*fl/fl*^ with *Col2α1-Cre*^*ERT*^ mice. Wild-type (WT) C57BL/6 J mice were obtained from Gempharmatech (Nanjing, Jiangsu, China). Animals were housed at the Experimental Animal Center of Tongji Hospital, Tongji Medical College, Huazhong University of Science and Technology. All experiments were approved by the Ethics Committee on Animal Experimentation of Tongji Hospital, Tongji Medical College, Huazhong University of Science and Technology (No. TJH-201809006). Ten-week-old *Mysm1*-Ctrl and *Mysm1*-cKO male mice were intraperitoneally administered with 30 mg/kg/day tamoxifen (10510-29-1, Sigma) for 5 days. DMM surgery was performed to induce OA in 12-week-old mice, as previously described.^47^ Briefly, the joint capsule of the right knees of the mice was opened, and the medial meniscus-tibial ligament was transected. For sham-operated mice, the joint was opened without ligament transection. For WT mice, one week post-surgery, 10 μL of Sh-*Mysm1*, Sh-*Control* (Sh-*Con*), AV-*Mysm1*, or AV-*Con* adenoviruses (1 × 10^9^ plaque-forming units [PFUs]) were injected into the right knee joint weekly for 8 weeks using a 31 G needle (Hamilton, Switzerland).

### Micro-computed tomography

All surgical mice were euthanized, and the right knees were harvested and fixed in 4% formaldehyde for 48 hours. High-resolution micro-computed tomography (μCT, Scanco Medical, Bassersdorf, Switzerland) was employed to examine osteophyte formation at a 15 µm resolution, utilizing 70 kVP and 112 µA x-ray energy. Three-dimensional joint images were reconstructed to assess osteophyte development around the joints, with osteophyte formation quantified on a scale of 0 to 3 in each group.^48^

### Culture of mouse primary chondrocytes and 293 T cells

Chondrocytes from WT or *Mysm1*-KO mice were cultured in vitro. Unless otherwise specified, WT chondrocytes were used in this study. *Mysm1*-KO chondrocytes were derived from *Mysm1*-KO mice, while CAG-negative mice served as controls. Briefly, knee joint cartilage was dissected from 5-day-old mouse pups and digested with 0.25% trypsin for 30 minutes. Subsequently, the cartilage was further digested with 0.25% collagenase II dissolved in Dulbecco’s Modified Eagle Medium (DMEM)/F12 (Hyclone, USA) for 5 hours. The cells were then cultured in DMEM/F12 supplemented with 10% fetal bovine serum (FBS) (Yeason, China), 100 units/mL penicillin, and 100 μg/mL streptomycin at 37 °C in a 5% CO_2_ environment. 293 T cells were cultured and passaged in DMEM with high glucose and 10% FBS. Chondrocytes or 293 T cells were plated on 10 cm plates and grown to approximately 80%–90% confluence before use.

### Plasmid mutation and Transfections

To manipulate RIPK2 phosphorylation levels, serine at the 176 site of RIPK2 was mutated to Aspartate (*Ripk2*^*S176D*^) or Alanine (*Ripk2*^*S176A*^). The *Ripk2*^*S176D*^ and *Ripk2*^*S176A*^ plasmids were synthesized by Tsingke (Beijing, China). For overexpression, chondrocytes or 293 T cells were transfected with 1 μg plasmid along with 5 μL of Lipofectamine 3000 and 250 μL of Opti-MEM in 2 mL of complete medium. For siRNA transfections, 50 pmol/L siRNA (5′-AGATGTTCCTAATAGTAAA-3′) targeting mouse *Mysm1* or (5′-GAAAGACCATCCTTTTTGA-3′) targeting mouse *Ripk2*, or scrambled siRNAs, 5 μL of Lipofectamine 3000, and 250 μL of Opti-MEM were mixed with 2 mL of complete medium and added to each well. After 48 hours of transfection, cells were harvested for subsequent treatments.

### RNA extraction and RT-qPCR

Total RNA was extracted using the TRIzol reagent (Takara, Japan). For cDNA synthesis, 0.5–1.0 μg of total RNA was used with the First Strand cDNA Synthesis Kit (Yeason, Shanghai, China). Subsequently, mRNA levels were quantified by amplifying the templates with SYBR Green (Yeason). All primers used are listed in Table S3. The relative mRNA levels of target genes were calculated *via* the 2^−ΔΔCt^ method.

### RNA sequencing

Whole-transcriptome sequencing was conducted on primary chondrocytes from *Mysm1*-KO and control mice, with three independent replicates per group. A total of 200 ng of total RNA from each cartilage sample was utilized for sequencing. Paired-end 2 × 100–bp RNA sequencing (Illumina TruSeq RNA Library Prep Kit, Illumina HiSeq2000, and Illumina HiSeq4000) was performed. The quality of raw reads was assessed using MultiQC.^49^ Differential expression analysis was carried out with the R package ‘DEseq2’. Genes with a |log_2_FC | >1 and an adjusted *P*-value of <0.05 (calculated using the moderated t-statistic with the Benjamini-Hochberg method to control the false discovery rate^50^) were considered DEGs. Enrichment analysis of DEGs was performed using the R package ‘clusterProfiler’, identifying significant GO and KEGG terms as those with a BH-adjusted *P*-value < 0.05.

### Western blot

Cartilage tissues from normal and OA patients were ground and lysed using ice-cold lysis buffer (Boster, China) containing 1% protease and phosphatase inhibitor (Boster, Wuhan, China) for 30 minutes. After centrifugation at 10 000 × *g* at 4 °C for 30 minutes, the supernatant was collected for Western blot analysis. Cultured chondrocytes or 293 T cells were also lysed on ice. Equal amounts of proteins were loaded onto SDS-PAGE (8%–15%) for electrophoresis. Nuclear and cytoplasmic proteins were extracted using a nuclear and cytoplasmic extraction kit (Boster). Proteins were transferred onto polyvinylidene fluoride (PVDF) membranes and blocked with 5% bovine serum albumin in Tris-buffered saline with 0.1% Tween 20 buffer (TBST) for 1 hour. After overnight incubation with primary antibodies at 4 °C, the membranes were incubated with horseradish peroxidase-conjugated secondary antibodies for 1 hour. Proteins were visualized with an enhanced chemiluminescence kit (Thermo Fisher Scientific, USA) using the ChemiDoc XRS System (Bio-Rad, USA). Protein quantification was performed using Image Lab software, with target proteins normalized to the relative total protein content.

### Co-immunoprecipitation (IP) and LC-MS/MS detection

Samples for co-immunoprecipitation were lysed using a Cell Lysis Buffer (Beyotime, Shanghai, China) for Western blotting and co-immunoprecipitation, containing 1% protease and phosphatase inhibitor (Boster). Primary antibodies or corresponding IgG, along with protein A/G beads (MedChemExpress), were added to the lysates for immunoprecipitation. The mixture was incubated overnight at 4 °C. Subsequently, the beads were rinsed with lysis buffer and boiled in loading buffer before subjecting the proteins to Western blot analysis. To identify MYSM1 binding proteins *via* mass spectrometry, chondrocytes were transfected with *Flag*-*Mysm1* plasmids. The proteins were then immunoprecipitated with an anti-Flag antibody overnight. Immunoprecipitated proteins were analyzed by SpecAlly Life Technology Co., Ltd (China). LC-MS/MS data acquisition was performed using a Q Exactive HF-X mass spectrometer coupled with an Easy-nLC 1200 system.

### Histology and immunohistochemistry (IHC) analysis

Cartilage tissues were soaked in 10% EDTA (pH 7.4) for 4 weeks, embedded in paraffin, and sectioned at 5 μm thickness. Following deparaffinization and hydration, Safranin O/Fast Green (S.O. Servicebio, China) and Toluidine Blue (T.B. Servicebio, China) staining were conducted as previously described.^51^ For S.O staining, the slides were incubated in Safranin O for 30 s followed by Fast Green staining for 1 h. For T.B staining, slides were immersed into Toluidine Blue solution for 5 minutes, After rinsing with water, slides were differentiated using 0.1% acetic acid for 2 minutes. Images were captured and assessed for the degree of articular cartilage damage using the Osteoarthritis Research Society International (OARSI) histopathology scoring system in a blinded manner.^52^ For IHC staining, sections were deparaffinized, hydrated, and blocked with 3% bovine serum albumin for 1 hour at room temperature, followed by overnight incubation with primary antibodies at 4 °C. Sections were then incubated with HRP-conjugated secondary antibodies and counterstained with hematoxylin.

### Immunofluorescence (IF)

Monolayer cultures of mouse chondrocytes were treated with 5 ng/mL IL-1β for various durations (15 minutes, 24 hours, or 48 hours). Following the treatment, chondrocytes were fixed in 4% paraformaldehyde, permeabilized with PBS containing 0.1% Triton X-100 for 10 minutes, and blocked with 1% BSA for 1 hour at room temperature. The cells were incubated with primary antibodies overnight at 4 °C, rinsed three times with PBS, and incubated with Alexa 488- or 543-conjugated secondary antibodies (1:1 000) for 1 hour at room temperature, protected from light. After washing with PBS, nuclei were counterstained with DAPI for 5 minutes. Correlation indexes were imaged using confocal microscopy (OLYMPUS FV1000, Japan). The catalog numbers and dilutions of the antibodies used in WB, IP, and IF experiments are provided in Table S4.

### Statistical analysis

Statistical analysis of the data was conducted using GraphPad Prism v. 5.0. Data are presented as means ± SEM. All experiments were independently repeated at least three times. One-way ANOVA followed by LSD’s post hoc test was used to assess differences among groups. Student’s t-test was applied to determine statistically significant differences between two groups. OARSI scores were analyzed using the Kruskal-Wallis test among all groups. A *P*-value of <0.05 was considered statistically significant.

## Supplementary information

Supplementary

## Data Availability

All data pertinent to the study are included in the article or provided as supplementary information.
