# Supplementary material for: MYSM1 attenuates osteoarthritis by recruiting PP2A to deubiquitinate and dephosphorylate RIPK2
Source: Bone Res. 2025 Jan 2;13:3. doi: 10.1038/s41413-024-00368-y (PMC11696715; doi:10.1038/s41413-024-00368-y)
Supplement: Supplementary file 1 — Supplementary [file 41413_2024_368_MOESM1_ESM.docx]

**Supplementary Information**

**MYSM1 attenuates osteoarthritis by recruiting PP2A to deubiquitinate and dephosphorylate RIPK2**

Kang Wei^1,2#^, Chuankun Zhou^1#^, Zixing Shu^1#^, Xingru Shang^1^, Yi Zou^1^, Wei Zhou^3^, Huanhuan Xu^3^, Yulin Liang^1^, Tian Ma^1^, Xuying Sun^1^*, Jun Xiao^1^*

1. Department of Orthopedics, Tongji Hospital, Tongji Medical College, Huazhong University of Science and Technology, 1095 Jiefang Ave, Wuhan, Hubei, China, 430030.

2. Department of Plastic Surgery, Zhongnan Hospital of Wuhan University, Wuhan, 430071, China, 430071.

3. Department of Obstetrics and Gynecology, Wuhan Children's Hospital, Tongji Medical College, Huazhong University of Science and Technology, 100 Xianggang Road, Wuhan, China,430000.

4. Institute of Hepatobiliary Diseases, Transplant Center, Hubei Key Laboratory of Medical Technology on Transplantation, Zhongnan Hospital of Wuhan University, 169 Donghu Road, Wuhan, China, 430071.

#These authors contributed equally

*Correspondence: Xuying Sun ([Xuying_Sun@hust.edu.cn](mailto:Xuying_Sun@hust.edu.cn)) or Jun Xiao ([Jun_Xiao@hust.edu.cn](mailto:Jun_Xiao@hust.edu.cn) )

**Fig. S1 MYSM1 regulates IL-1β induced cartilage degradation *in vitro*.**

**Fig. S2 Deletion of *Mysm1*** **promoted cartilage degeneration in mouse chondrocytes and *in vivo*.**

**Fig. S3 Overexpression *Mysm1* alleviated cartilage degeneration in mouse chondrocytes and *in vivo*.**

**Fig. S4 MYSM1 regulated NF-κB and MAPK signaling pathways.**

**Fig. S5 RIPK2 accelerated chondrocyte degeneration and activated NF-κB and MAPK signaling pathways.**

**Fig. S6 MYSM1 deactivated NF-κB and MAPK signaling pathways induced by RIPK2.**

**Fig. S7 p-RIPK2^S176D^ activated** **NF-κB and MAPK signaling pathways.**

**Fig. S8 p-RIPK2^S176D^ activated NF-κB and MAPK signaling pathways.**

**Fig. S9 MYSM1 was unable to deactivate NF-κB and MAPK signaling pathways after RIPK2^S176D^ mutation.**

**Supplementary Table. 1 Human sample information.**

**Supplementary Table. 2 MYSM1 positive cells (%).**

**Supplementary Table. 3 Primer sequences for RT-PCR.**

**Supplementary Table. 4 Antibodies information.**

**
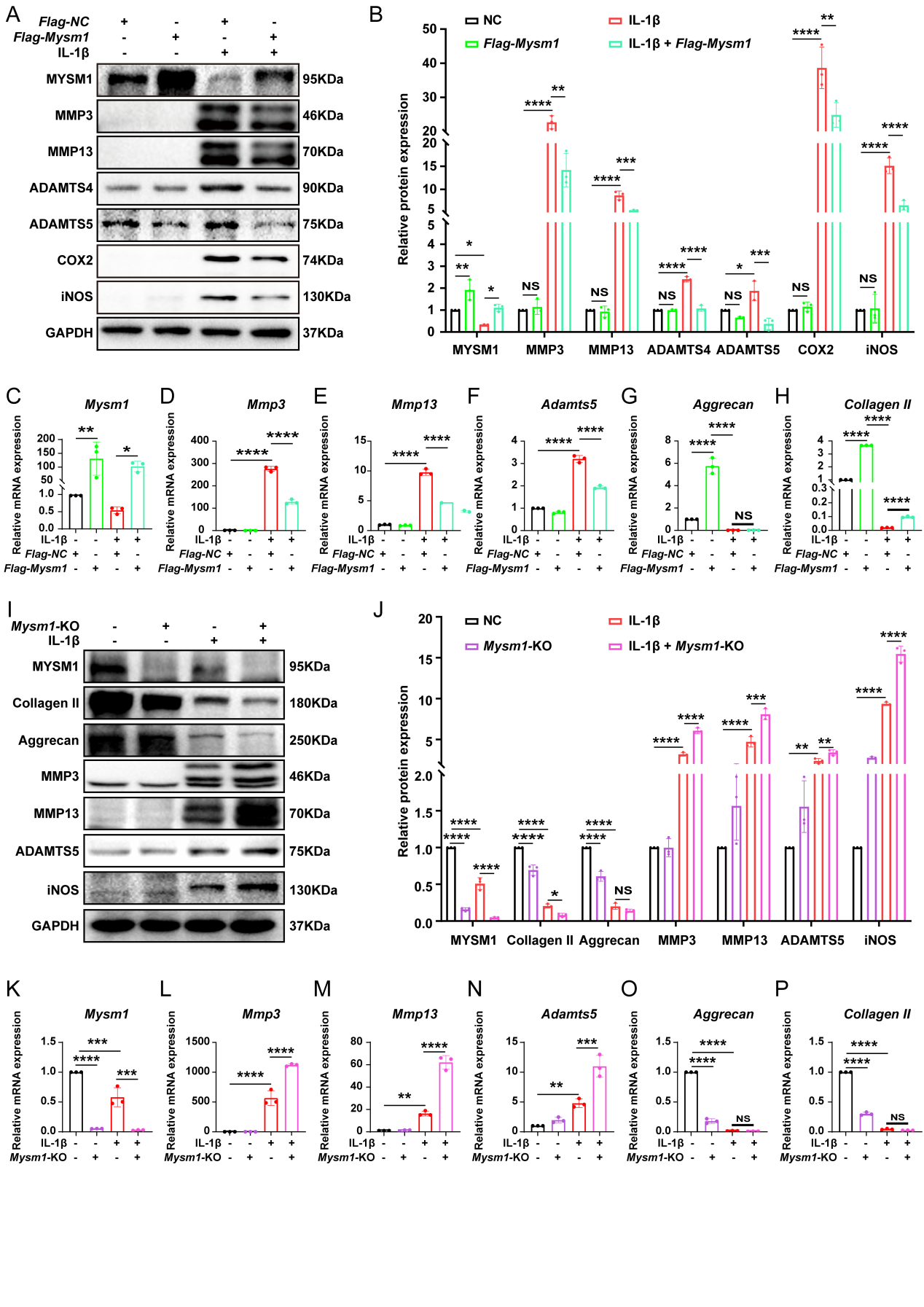
**

**Fig. S1 MYSM1 regulates IL-1β induced cartilage degradation *in vitro*.** *Mysm1* was overexpressed in mouse chondrocytes for 48 h, then the cells were further treated with or without 5 ng/ml IL-1β for 24 h. (A) Representative western blot of MYSM1, MMP3, MMP13, Adamts4, Adamts5, COX2, and iNOS and (B) its quantitative analysis, n=3. RT-PCR results showed mRNA levels of *Mysm1* (C), *Mmp3* (D), *Mmp13* (E), *Adamts5* (F), *Aggrecan* (G) and *CollagenⅡ* (H) with or without IL-1β treatment for 48 h, n=3. Primary chondrocytes from *Mysm1*-KO or wildtype were cultured, then the cells were exposed to 5 ng/ml IL-1β for 48 h or not. (I) Representative western blot of MYSM1, CollagenⅡ, Aggrecan, MMP3, MMP13, Adamts5, and iNOS and (J) its quantitative analysis, n=3. RT-PCR results showed mRNA levels of *Mysm1* (K), *Mmp3* (L), *Mmp13* (M), *Adamts5* (N), *Aggrecan* (O), Collagen Ⅱ (P), n=3. All data were presented as mean ± SD. *p < 0.05, **p < 0.01, ***p < 0.001, ****p<0.0001.


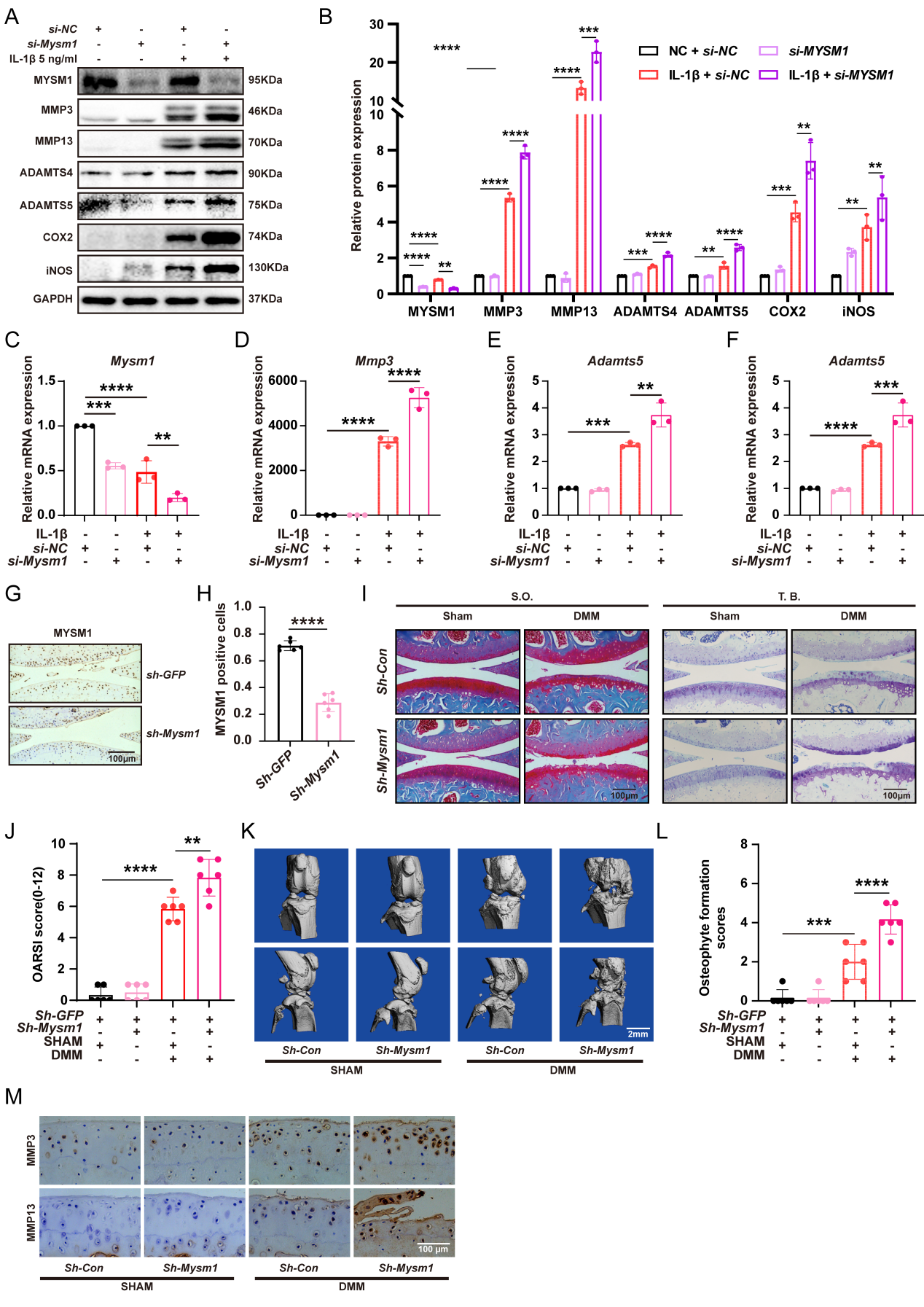


**Fig. S2** **Deletion of *Mysm1*** **promoted cartilage degeneration in mouse chondrocytes and *in vivo*.** Cultured mouse chondrocytes were transfected with si-NC or si-*Mysm1* for 48 h, then 5 ng/ml IL-1β was applied to treat the chondrocytes for 24 h. (A) Representative western blots of MYSM1, MMP3, MMP13, Admats4, Adamts5, COX2, iNOS, and (B) its quantitative analysis, n=3. (C-F) RT-PCR results showed mRNA levels, n=3. (G) DAB staining of MYSM1 in *Sh-GFP* or *Sh*-*Mysm1* viruses injected mice 8 weeks post DMM surgery. Scale bar = 100 µm. (H) Quantitative analysis of MYSM1 in G, n=6. (I) S.O. and T.B. staining and (J) OARSI grades of knee joints, n=6. (K) Representative MicroCT images of the knee joints in each group. Scale bar=2.0 mm. (L) Osteophyte formation scores of K, n=6. (M) IHC staining of MMP3 and MMP13 in the joint cartilage of *Sh-GFP* or *Sh-Mysm1* viruses Scale bar = 100 µm. All data were presented as mean ± SD. *p < 0.05, **p < 0.01, ***p < 0.001, ****p<0.0001.

**
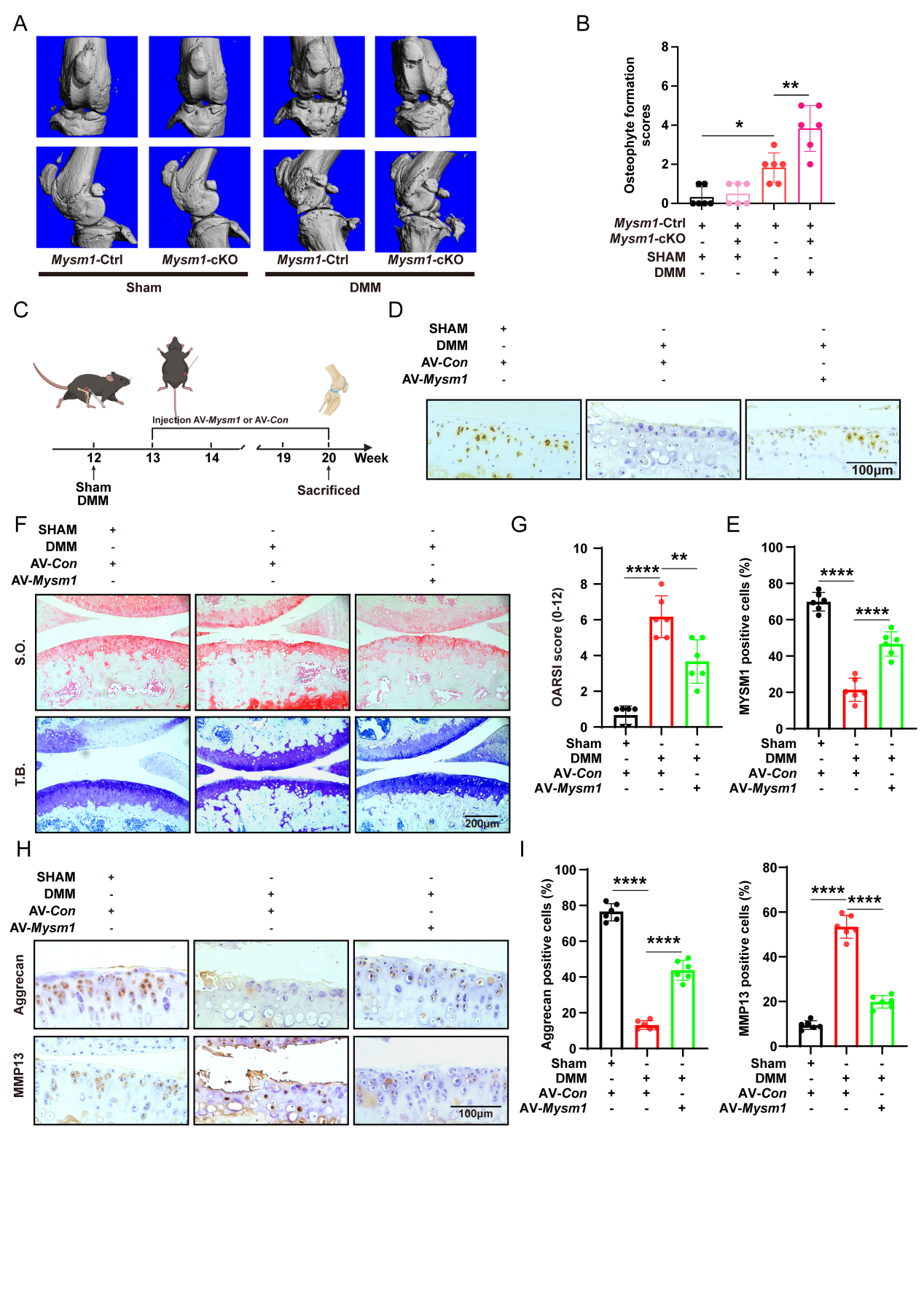
**

**Fig. S3 Overexpression *Mysm1* alleviated cartilage degeneration in mouse chondrocytes and *in vivo*.** (A) Representative MicroCT images of the knee joints in *Mysm1*-cKO or *Mysm1*-Ctrl mice. Scale bar = 1mm. (B) Osteophyte formation scores of A, n=6. (C) The experimental design schematic illustration of AV-*Mysm1* or AV-*Con* administration and DMM surgery for mice drawn by BioRender. (D) IHC staining of MYSM1 in mice cartilage 8 weeks after AV-*Mysm1* or AV-*Con* administration, scale bar = 100 µm. (E) Quantitative analysis of MYSM1 in B, n=6. (F) H.E., S.O. and T.B. staining and (G) OARSI grade of knee joints from mice after AV-*Mysm1* or AV-*Con* administration. Scale bars=200 µm, n=6. (H) IHC staining of Aggrecan and MMP13 staining images. Scale bar = 100 µm, n=6. (I) Quantification of H, n=6. All data were presented as mean ± SD. *p < 0.05, **p < 0.01, ****p<0.0001.

**
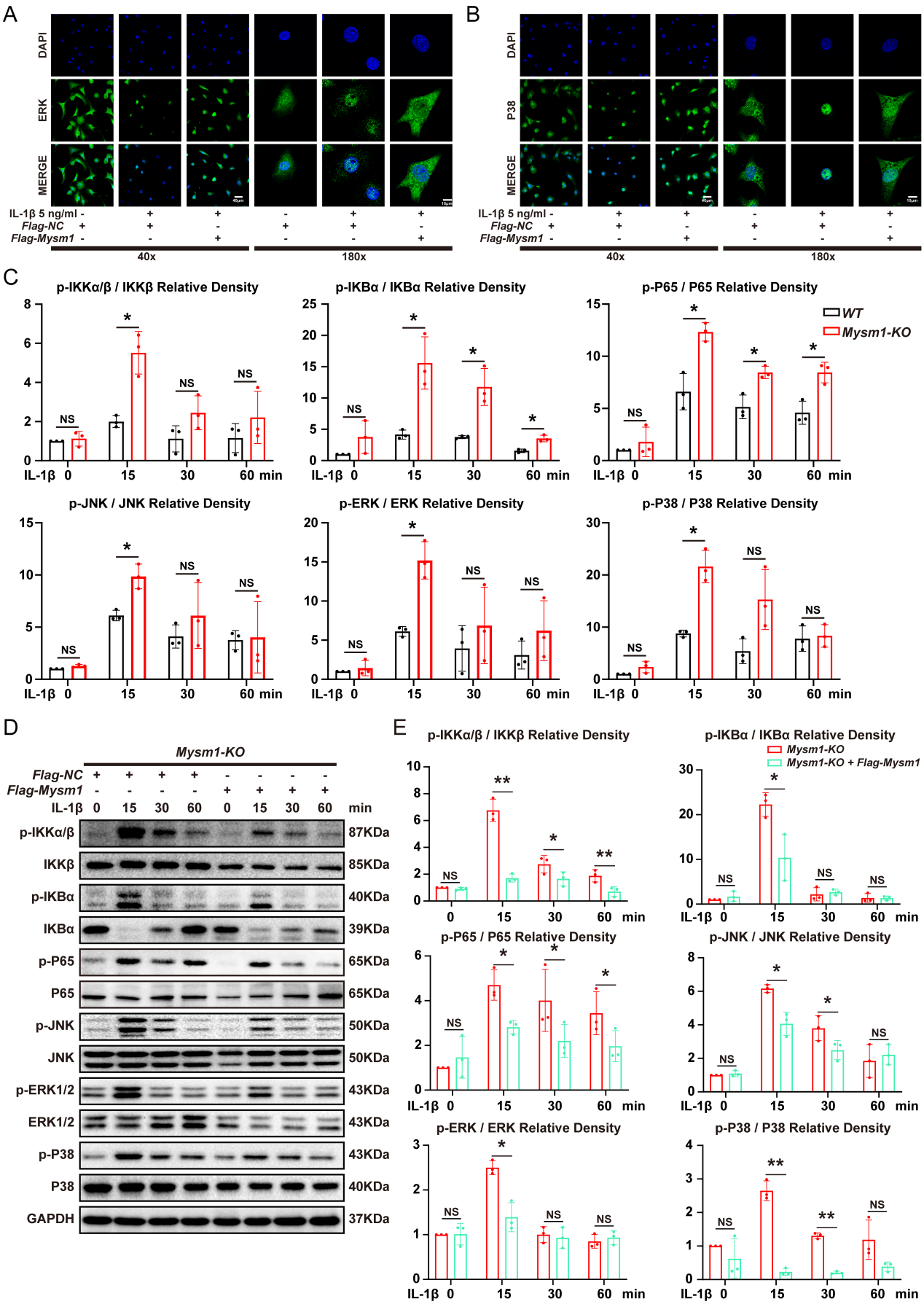
**

**Fig. S4 MYSM1 regulated NF-κB and MAPK signaling pathways.** (A) Immunofluorescence staining of ERK in primary mouse chondrocytes after treatment with 5 ng/ml IL-1β for 15 min. scale bar = 40 and 10 µm. (B) Immunofluorescence staining of P38 in primary mouse chondrocytes after treatment with 5 ng/ml IL-1β for 15 min. scale bar = 40 and 10 µm. (C) The phosphorylation ratio of Fig. 4G-H. *Msym1-KO* chondrocytes were transfected with *Mysm1* plasmids for 48 h, then 5 ng/ml IL-1β was applied to treat the chondrocytes for indicated time points. (D) Representative western blots of NF-κB and MAPK signaling pathways, and (E) the phosphorylation ratio, n=3. All data were presented as mean ± SD. *p < 0.05, **p < 0.01.


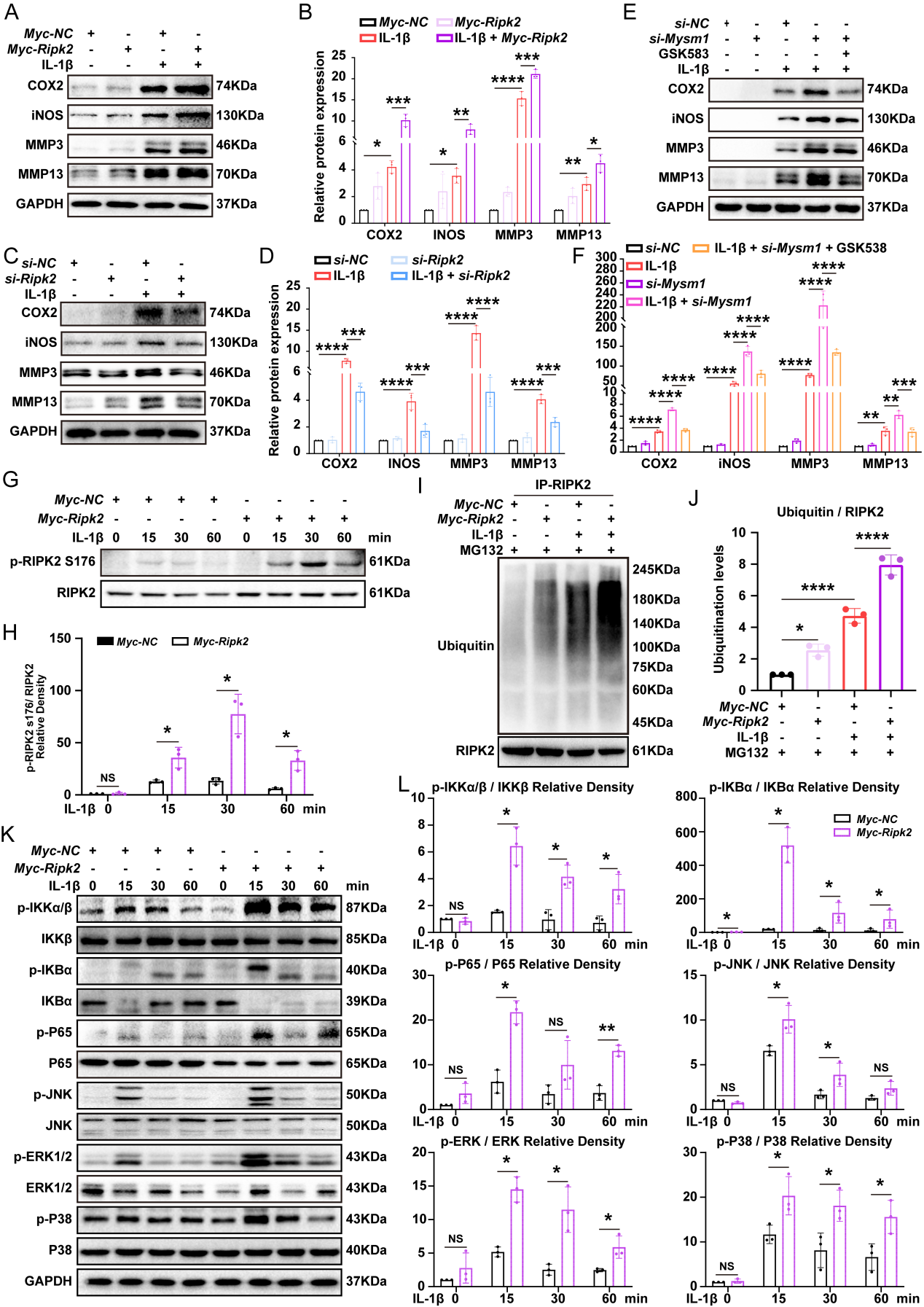


**Fig. S5 RIPK2 accelerated chondrocyte degeneration and activated NF-κB and MAPK signaling pathways.** (A) Representative western blots of MMP3, MMP13, COX2 and iNOS in chondrocytes after *Ripk2* overexpression, and (B) its quantitative analysis, n=3. (C) Representative western blots of MMP3, MMP13, COX2 and iNOS in mouse chondrocytes after *Ripk2* knockout, and (D) its quantitative analysis, n=3. (E) Representative western blots of MMP3, MMP13, COX2 and iNOS after transfecting with *si-Mysm1* then 5 ng/ml IL-1β was applied to treat the mouse chondrocytes for 24 h with or without RIPK2 antagonist GSK583 preincubation for 30 min, and (F) its quantitative analysis, n=3. (G) Western blots of p-RIPK2 and RIPK2 in mouse chondrocytes after overexpressing *Ripk2* and (H) the phosphorylation ratio. (I) Immunoprecipitation assay using anti-RIPK2 antibody to detect ubiquitination of RIPK2 after *Ripk2* overexpression with 5 ng/ml IL-1β treatment in mouse chondrocytes, and (J) Quantification analysis of RIPK2 ubiquitination levels, n=3. (K) Representative western blot of NF-κB and MAPK signaling pathways in mouse chondrocytes after overexpressing *Ripk2* and (L) the phosphorylation ratio, n=3. All data were presented as mean ± SD. *p < 0.05, **p < 0.01, ***p < 0.001, ****p<0.0001.


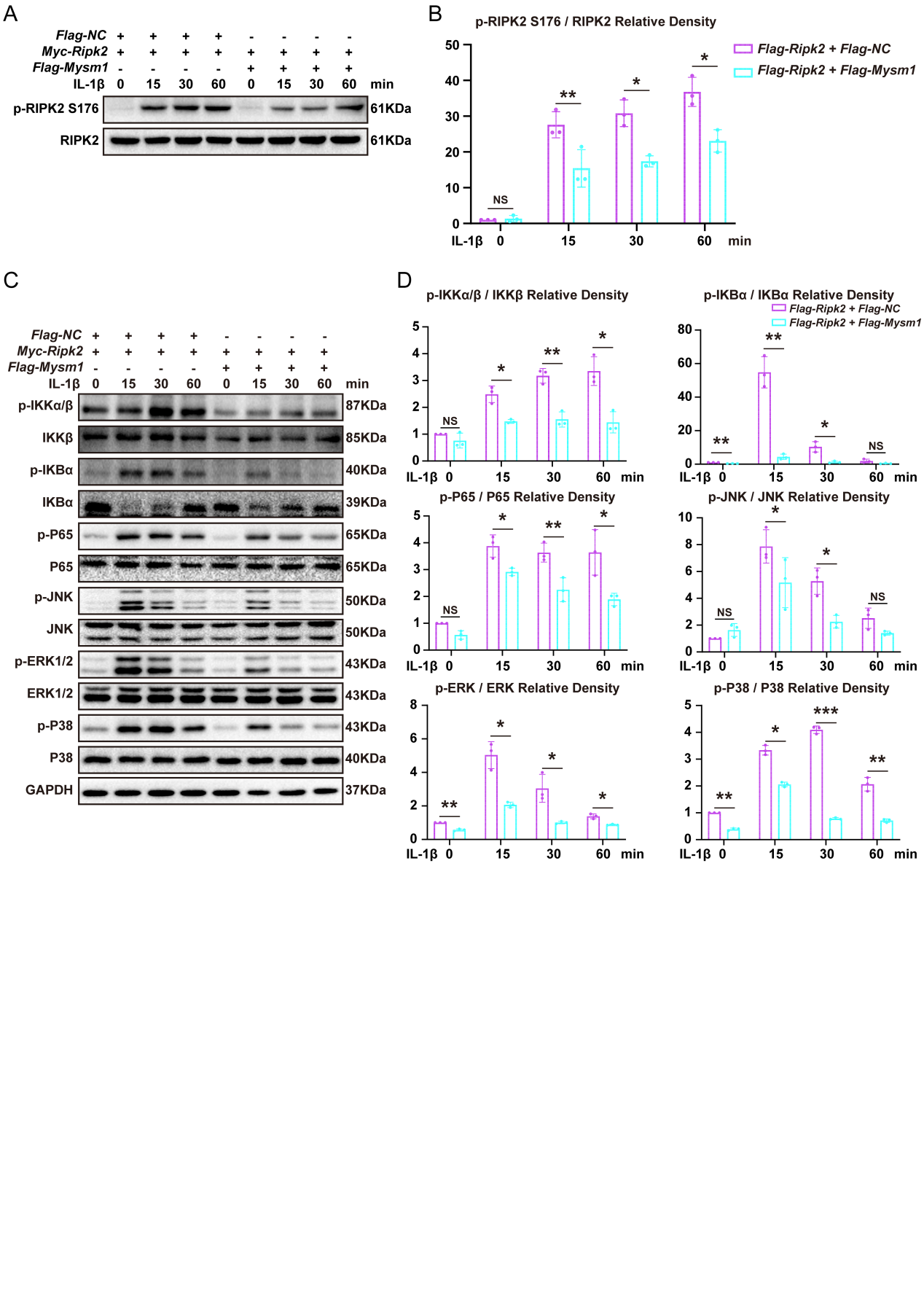


**Fig. S6 MYSM1 deactivated NF-κB and MAPK signaling pathways induced by RIPK2.** Cultured mouse chondrocytes were transfected with *Mysm1* or *Ripk2* plasmids for 48 h, 5 ng/ml IL-1β was applied to treat the chondrocytes for indicated time points. (A) Western blots of p-RIPK2 and RIPK2 and (B) the phosphorylation ratio, n=3. (C) Western blots of NF-κB and MAPK signaling pathways, and (D) the phosphorylation ratio, n=3. *p < 0.05, **p < 0.01, ***p < 0.001.


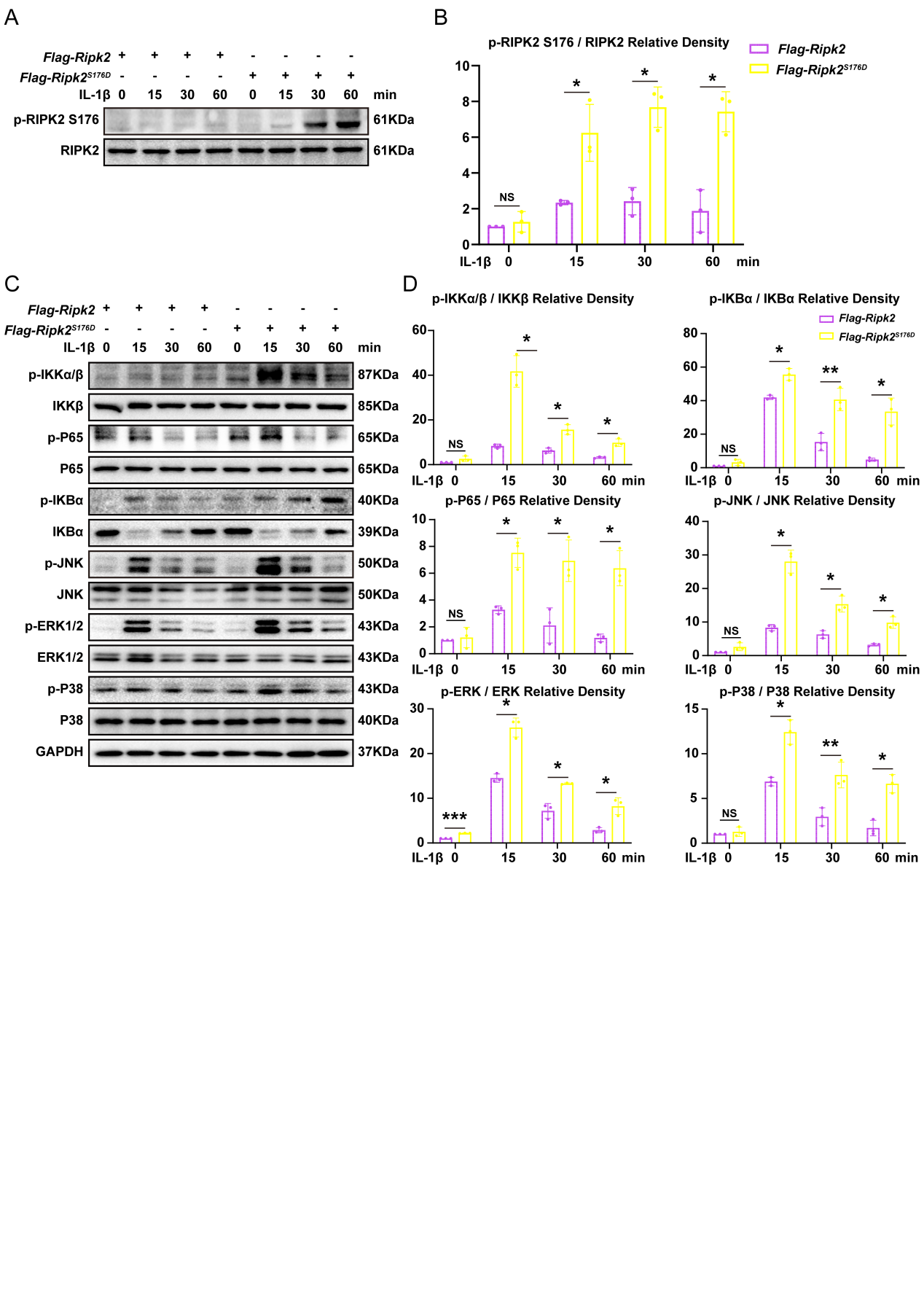


**Fig. S7 p-RIPK2^S176D^ activated** **NF-κB and MAPK signaling pathways.** Cultured mouse chondrocytes were transfected with *Ripk2* or *Ripk2^S176D^* plasmids for 48 h, 5 ng/ml IL-1β was applied to treat the chondrocytes for indicated time points. (A) Western blots of p-RIPK2 and RIPK2 and (B) the phosphorylation ratio, n=3. (C) Western blots of NF-κB and MAPK signaling pathways, and (D) the phosphorylation ratio, n=3. *p < 0.05, **p < 0.01.


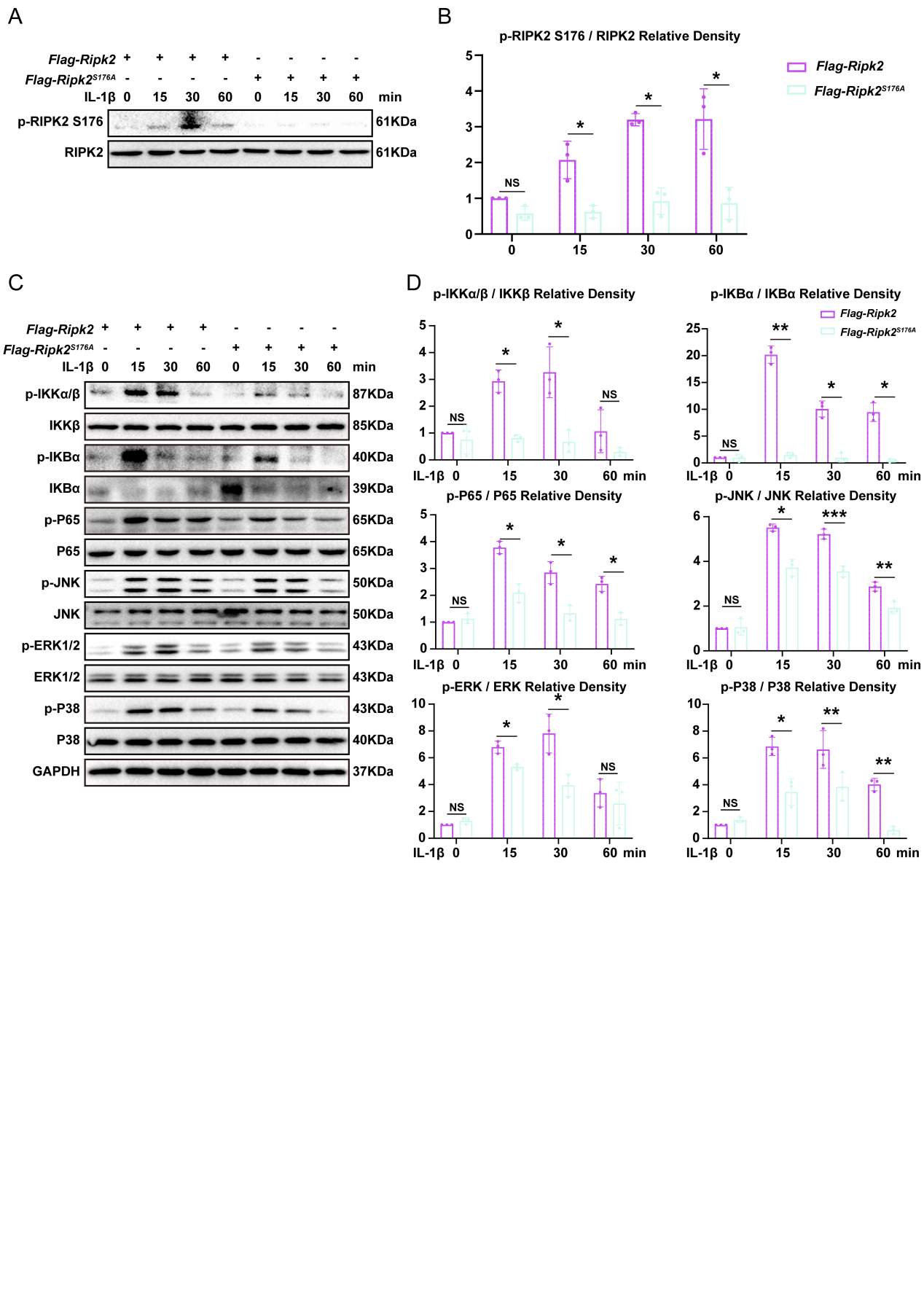


**Fig. S8 RIPK2^S176A^ failed to activate NF-κB and MAPK signaling pathways.** Cultured chondrocytes were transfected with *Ripk2* or *Ripk2^S176A^* plasmids for 48 h, 5 ng/ml IL-1β was applied to treat the chondrocytes for indicated time points. (A) Western blots of p-RIPK2 and RIPK2 and (B) the phosphorylation ratio, n=3. (C) Western blots of NF-κB and MAPK signaling pathways, and (D) the phosphorylation ratio, n=3. *p < 0.05, **p < 0.01.


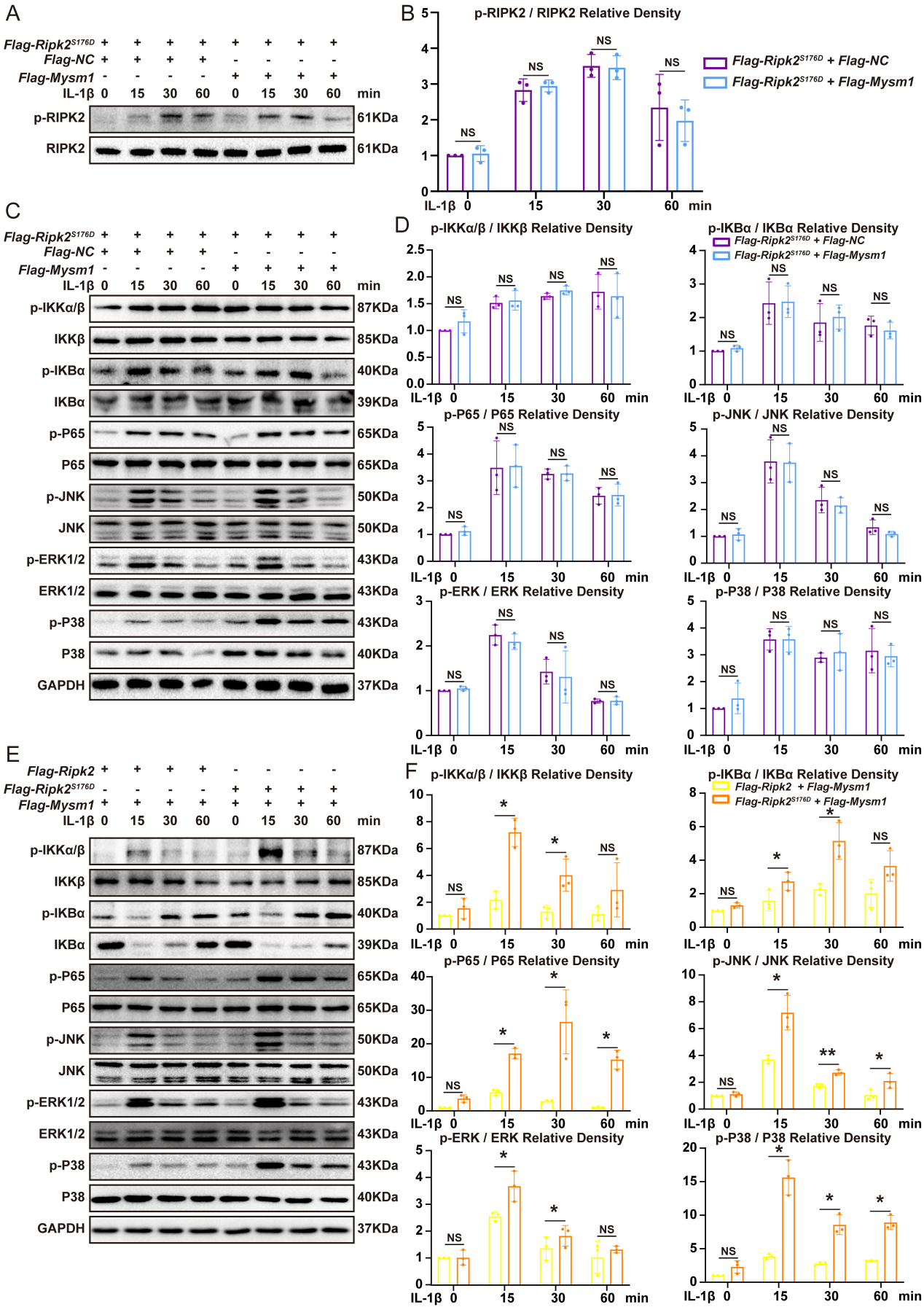


**Fig. S9 MYSM1 was unable to deactivate NF-κB and MAPK signaling pathways after RIPK2^S176D^ mutation.** Mouse chondrocytes were transfected with *Ripk2**^S176D^* or *Mysm1* plasmids and 5 ng/ml IL-1β was applied to treat the chondrocytes for indicated time points (0, 15, 30, 60 min). (A) Western blots of p-RIPK2 and RIPK2 and (B) the phosphorylation ratio. (C) Representative western blot of NF-κB and MAPK signaling pathways and (D) the phosphorylation ratio, n=3. Mouse chondrocytes were co-transfected with *Mysm1* and *Ripk2* or *Ripk2^S176D^* (E) Representative western blot of NF-κB and MAPK signaling pathways, and (F) the phosphorylation ratio, n=3. All data were presented as mean ± SD. *p < 0.05, **p < 0.01.

**Supplementary Table. 1 Human sample information.**

| **Number** | **Age** | **Gender** | **Diagnose** | **Apply** |
| --- | --- | --- | --- | --- |
| NC1 | 57 years | Male | Cerebral hernia | WB |
| NC2 | 39 years | Female | Cerebral hemorrhage | WB and IF |
| NC3 | 56 years | Male | Cerebral hemorrhage | WB |
| NC4 | 43 years | Male | Cerebral hemorrhage | IF |
| NC5 | 40 years | Male | Cerebral hemorrhage | IF |
| NC6 | 47 years | Female | Cerebral hemorrhage | IF |
| OA1 | 65 years | Female | Osteoarthritis | WB |
| OA2 | 64 years | Female | Osteoarthritis | WB and IF |
| OA3 | 77 years | Female | Osteoarthritis | WB |
| OA4 | 59 years | Male | Osteoarthritis | WB and IF |
| OA5 | 61 years | Female | Osteoarthritis | WB |
| OA6 | 64 years | Male | Osteoarthritis | IF |
| 0A7 | 69 years | Female | Osteoarthritis | WB |
| OA8 | 63 years | Female | Osteoarthritis | WB |
| OA9 | 74 years | Female | Osteoarthritis | IF |
| OA10 | 67 years | Female | Osteoarthritis | IF |

**Supplementary Table. 2** **MYSM1 positive cells (%).**

| Number | MYSM1 positive cells (%) | Mean | Student's t test |
| --- | --- | --- | --- |
| NC2 | 68.42% | **59.96%** | p = 0.0002 |
| NC4 | 51.11% |  |  |
| NC5 | 66.67% |  |  |
| NC6 | 53.66% |  |  |
| OA2 | 9.62% | **17.26%** |  |
| OA4 | 5.56% |  |  |
| OA6 | 20.41% |  |  |
| OA9 | 26.19% |  |  |
| OA10 | 24.53% |  |  |

**Supplementary Table. 3 Primer sequences for RT-PCR.**

| **Gene** |  | **Primer sequence** |
| --- | --- | --- |
| ***Mysm1*** | F | TGCACAGCCGGGAAATGAT |
|  | R | ATGGTGCTATCCAGAGTCCAA |
| ***Mmp3*** | F | ACATGGAGACTTTGTCCCTTTTG |
|  | R | TTGGCTGAGTGGTAGAGTCCC |
| ***Mmp13*** | F | CTTCTTCTTGTTGAGCTGGACTC |
|  | R | CTGTGGAGGTCACTGTAGACT |
| ***Adamts5*** | F | GGAGCGAGGCCATTTACAAC |
|  | R | CGTAGACAAGGTAGCCCACTTT |
| ***CollagenⅡ*** | F | GGGAATGTCCTCTGCGATGAC |
|  | R | GAAGGGGATCTCGGGGTTG |
| ***Aggrecan*** | F | AGGTGTCGCTCCCCAACTAT |
|  | R | CTTCACAGCGGTAGATCCCAG |
| ***Gapdh*** | F | AGGTCGGTGTGAACGGATTTG |
|  | R | TGTAGACCATGTAGTTGAGGTC |

**Supplementary Table. 4 Antibodies information.**

| Antibody | Brand | Application and Dilution |
| --- | --- | --- |
| ADAMTS4 | Abcam, ab185722 | WB: 1/1000 |
| ADAMTS5 | Boster, A02802-1 | WB: 1/1000 |
| Aggrecan | Abclonal, A8536 | WB: 1/1000, IHC: 1/100 |
| Collagen II | Proteintech, 28459-1-AP | WB: 1/1000, IHC: 1/100 |
| COX2 | Abclonal, A1253 | WB: 1/1000 |
| iNOS | Abclonal, A3774 | WB: 1/1000 |
| GAPDH | Proteintech, 60004-1-Ig | WB: 1/10000 |
| MMP13 | Proteintech, 18165-1-AP | WB: 1/1000, IHC: 1/100 |
| MMP3 | Proteintech, 66338-1-Ig | WB: 1/1000, IHC: 1/100 |
| MYSM1 | Abcam, ab193081 | WB: 1/1000, IP: 1/100 |
| MYSM1 | Proteintech, 20078-1-AP | IF: 1/100 |
| p-IKKα/β | CST, 2697 | WB: 1/1000 |
| IKKβ | CST, 8943 | WB: 1/1000 |
| p-IKB | CST, 2859 | WB: 1/1000 |
| IKB | CST, 4812 | WB: 1/1000 |
| p-P65 | CST, 8242 | WB: 1/1000 |
| P65 | CST, 3033 | WB: 1/1000, IF: 1/100 |
| p-JNK | CST, 4668 | WB: 1/1000 |
| JNK | CST, 9252 | WB: 1/1000, IF: 1/100 |
| p-ERK1/2 | CST, 4370 | WB: 1/1000 |
| ERK1/2 | CST, 4695 | WB: 1/1000, IF: 1/100 |
| p-P38 | CST, 9211 | WB: 1/1000 |
| P38 | CST, 8690 | WB: 1/1000, IF: 1/100 |
| RIPK2 | Santa, sc-136059 | WB: 1/1000, IP:1:50, IF: 1/100 |
| p-RIPK2 S531 | Affinity Biosciences, AF7118 | WB: 1/1000 |
| p-RIPK2 Y381 | Affinity Biosciences, AF7119 | WB: 1/1000 |
| p-RIPK2 S176 | Affinity Biosciences, AF0049 | WB: 1/1000, IF: 1/100 |
| Ubiquitin | CST, 3936 | WB: 1/1000 |
| PP2Ac | Billerica, SAB4200266 | WB: 1/1000 |
| p-PP2Ac(pY307) | Abcam, ab314196 | WB: 1/1000 |
| Myc | Abclonal, AE010 | WB: 1/1000, IP:1:100 |
| Flag | Abclonal, AE005 | WB: 1/1000, IP:1:100 |
| HA | Proteintech, 81290-1-RR | WB: 1/1000, IP:1:100 |
| Lamin-B1 | Proteintech, 12987-1-AP | WB: 1/5000 |
